# Safety and Efficacy of Brolucizumab in the Treatment of Patients with Diabetic Macular Edema and Diabetic Retinopathy: A Systematic Review and Meta-Analysis

**DOI:** 10.3390/jcm15176518

**Published:** 2026-08-23

**Authors:** Sharifa N. Bourisly, Fatmah S. Semairan, Yousef Mesaed Al-Shammari, Noor Alali, Lulwa Abbas Alfoudari, Abdulaziz F. Abdulkareem, Abdullah M. Alharran

**Affiliations:** 1 Mubarak Alkabeer Hospital, Ministry of Health, P.O. Box 5, Safat, Kuwait City 13001, Kuwait; 2 Farwaniya Hospital, Ministry of Health, P.O. Box 5, Safat, Kuwait City 13001, Kuwait; 3 Department of Ophthalmology, Al-Bahar Eye Center, Ibn Sina Hospital, Ministry of Health, P.O. Box 5, Safat, Kuwait City 13001, Kuwait; 4 Aljahra Hospital, Ministry of Health, P.O. Box 5, Safat, Kuwait City 13001, Kuwait; 5 College of Medicine and Medical Sciences, Arabian Gulf University, Salmaniya, Manama P.O. Box 26671, Bahrain

**Keywords:** brolucizumab, diabetic macular edema, diabetic retinopathy, proliferative diabetic retinopathy, anti-VEGF

## Abstract

**Background:** Brolucizumab is an intravitreal anti-vascular endothelial growth factor (anti-VEGF) agent developed to improve retinal drying and treatment durability in diabetic eye disease. However, its overall efficacy and safety in diabetic macular edema (DME) and diabetic retinopathy remain uncertain because available trials differ in population, comparator, dosing schedule, and follow-up. **Methods:** We searched PubMed, Cochrane Library, Scopus, and Web of Science from inception to 25 April 2026. Randomized controlled trials evaluating intravitreal brolucizumab in adults with DME, diabetic retinopathy, or proliferative diabetic retinopathy (PDR) were included. Comparators were aflibercept, panretinal photocoagulation, or other active/conventional treatments. The main efficacy outcomes were change in best-corrected visual acuity (BCVA) and central subfield thickness/central subfield foveal thickness (CST/CSFT). Safety outcomes included ocular, non-ocular, serious ocular, and serious non-ocular adverse events. Random-effects meta-analyses were performed. **Results:** Five reports representing four phase 3 randomized controlled trials were included, enrolling 2132 participants overall; the four-trial primary 6 mg analyses included 1942 participants. Brolucizumab was not associated with a significant improvement in BCVA compared with control treatment (mean difference [MD], 1.21 letters; 95% confidence interval [CI], −1.12 to 3.54; *p* = 0.3; I^2^ = 85.1%). However, brolucizumab significantly reduced CST/CSFT (MD −24.45 µm, 95% CI −47.43 to −1.47; *p* = 0.0370; I^2^ = 78.2%). No statistically significant differences were detected between groups in the assessed safety outcomes, including ocular adverse events (risk ratio [RR], 0.88), non-ocular adverse events (RR 1.00), serious ocular adverse events (RR 0.63), and serious non-ocular adverse events (RR 0.89). Separate analyses of brolucizumab-specific ocular events showed no statistically significant differences for intraocular inflammation (RR 2.35, 95% CI 0.36–15.42), retinal vasculitis (RR 2.10, 95% CI 0.22–20.10), or retinal vascular occlusion (RR 2.13, 95% CI 0.46–9.87); however, estimates were imprecise because of the small number of events. **Conclusions**: Across the four included trials, brolucizumab was not associated with a statistically significant difference in BCVA compared with control treatment. In the three DME trials, brolucizumab achieved a greater reduction in CST/CSFT. It may be useful for selected DME patients, while its role in PDR requires longer-term evidence.

## 1. Introduction

Diabetes mellitus (DM) is increasing worldwide, and diabetic retinopathy (DR) remains one of its most important microvascular complications and a major cause of preventable visual loss in working-age adults [1,2]. Approximately 22% of people with DM have DR, and more than 6% have vision-threatening disease, creating a large and growing clinical burden for patients, clinicians, and health systems [3]. Vision loss in DR usually occurs through diabetic macular edema (DME), proliferative diabetic retinopathy (PDR), or both and these complications may lead to irreversible functional impairment if treatment is delayed [4,5]. DME is characterized by retinal vascular leakage and thickening at the macula, while PDR reflects advanced retinal ischemia with pathologic neovascularization and risk of vitreous hemorrhage or tractional retinal detachment [5,6]. Over the past two decades, intravitreal anti-vascular endothelial growth factor (anti-VEGF) therapy has become central to the management of center-involved DME and has reduced reliance on focal or grid laser therapy [7,8]. Anti-VEGF therapy is also an effective option for PDR, although panretinal photocoagulation (PRP) remains a long-established treatment and is still widely used when adherence, access, or cost are concerns [9]. Despite these advances, repeated injections, frequent monitoring, incomplete anatomic response, and variable durability continue to limit long-term disease control. Additionally, anti-VEGF therapies are effective only in approximately half of patients with DME [10]. Therefore, newer anti-VEGF agents are being evaluated to maintain visual outcomes while improving retinal drying and reducing treatment burden.

Brolucizumab is a single-chain antibody fragment that binds vascular endothelial growth factor A (VEGF-A) with high affinity and has a low molecular weight, allowing a high molar dose to be delivered by intravitreal injection [11]. In DME, the KESTREL and KITE phase 3 trials showed that brolucizumab produced robust visual gains and anatomic improvements compared with aflibercept, with durable outcomes through longer follow-up [12,13]. The KINGFISHER trial further compared brolucizumab with aflibercept at equal 4-week dosing intervals and suggested comparable visual efficacy with favorable anatomic effects [14]. More recently, the CONDOR trial evaluated brolucizumab against PRP in PDR and reported noninferior and superior visual acuity outcomes at week 54 [2]. However, the clinical use of brolucizumab requires balanced interpretation because intraocular inflammation, retinal vasculitis, and retinal vascular occlusion have been reported as important safety concerns. Individual trials provide useful data, but their differences in population, comparator, dosing schedule, follow-up duration, and outcome reporting make the overall benefit-risk profile difficult to judge from single studies alone. Therefore, this systematic review and meta-analysis aimed to synthesize randomized evidence on the efficacy and safety of brolucizumab in patients with DME and DR, focusing on visual, anatomic, ocular safety, and systemic safety outcomes.

## 2. Methods

### 2.1. Study Design

This systematic review and meta-analysis was conducted in accordance with the PRISMA 2020 statement and the Cochrane Handbook for Systematic Reviews of Interventions [15,16]. The review question was defined according to the population, intervention, comparator, and outcome (PICO) framework. We evaluated adult patients with DME, DR, or PDR who received intravitreal brolucizumab and compared them with patients who received active or conventional comparator treatment. The protocol was registered in the International Prospective Register of Systematic Reviews (PROSPERO; CRD420261393885).

### 2.2. Data Sources and Search Strategy

PubMed, Cochrane Library, Scopus, and Web of Science were searched from inception to 25 April 2026, and after finishing, we re-checked the databases for any new eligible studies. The search strategy combined terms related to brolucizumab, including “brolucizumab” and “Beovu,” with terms related to diabetic eye disease, including “diabetic macular edema,” “diabetic retinopathy,” “proliferative diabetic retinopathy,” and related synonyms. The full search strategy and record yield from each database are presented in Supplementary Table S1.

### 2.3. Eligibility Criteria

We included randomized controlled trials (RCTs) evaluating intravitreal brolucizumab in adult patients with DME, DR, or PDR. Eligible comparator groups included aflibercept, PRP, or other active/conventional comparator treatments. Studies were required to report at least one extractable efficacy or safety outcome. We excluded non-randomized studies, reviews, editorials, conference abstracts without sufficient extractable data, duplicate reports without unique outcome data, and studies with ineligible populations, interventions, or comparators.

### 2.4. Study Selection

Records were de-duplicated before screening using EndNote 21 (Clarivate, Philadelphia, PA, USA) [17]. Titles and abstracts were screened first, followed by full-text assessment of potentially eligible reports. All screening steps were conducted by two independent authors using the Rayyan web application (Rayyan Systems Inc., Cambridge, MA, USA; accessed 25 April 2026) [18]. When more than one report described the same trial, data were extracted from the most relevant report for each outcome, while avoiding duplicate counting of patients.

### 2.5. Data Extraction

Data were extracted using a standardized form by two independent authors, with disagreements resolved by discussion until consensus was reached. Extracted items included study identity, design, country, study duration, follow-up, eligibility criteria, intervention details, comparator details, sample size, baseline characteristics, efficacy outcomes, and safety outcomes. For the primary meta-analysis, only the brolucizumab 6 mg arm of KESTREL was included. The 3 mg and 6 mg arms were combined only in the sensitivity analysis to avoid double-counting the shared aflibercept comparator group. When outcome data were presented only graphically, numerical values were extracted from the published figures using WebPlotDigitizer [19].

### 2.6. Outcome Measures

The main efficacy outcomes were change in best-corrected visual acuity (BCVA) at week 52 in the DME trials and at week 54 in the CONDOR trial, and change in central subfield thickness or central subfield foveal thickness (CST/CSFT) at week 52 or week 100, depending on the available trial report. Safety outcomes were extracted at the corresponding reported follow-up timepoint for each trial. Safety outcomes included at least one ocular adverse event, at least one non-ocular adverse event, at least one serious ocular adverse event, and at least one serious non-ocular adverse event. Extra-safety outcomes included intra-ocular inflammation, retinal vasculitis, and retinal vessel occlusion.

### 2.7. Risk of Bias and Certainty Assessment

Risk of bias for the included RCTs was assessed using the Cochrane Risk of Bias 2 (RoB 2) tool across the standard five domains: randomization process, deviations from intended interventions, missing outcome data, outcome measurement, and selection of the reported result [20]. Each study was judged as low risk, some concerns, or high risk of bias. Certainty of evidence for the main outcomes was evaluated using the Grading of Recommendations Assessment, Development and Evaluation (GRADE) approach [21]. The certainty rating for each outcome was judged across risk of bias, inconsistency, indirectness, imprecision, and other considerations. The final certainty of evidence was categorized as high, moderate, low, or very low.

### 2.8. Statistical Analysis

Meta-analysis was performed using mean differences (MDs) with 95% confidence intervals (CIs) for continuous outcomes and risk ratios (RRs) with 95% CIs for dichotomous outcomes. Random-effects models were used for all pooled analyses because clinical and methodological heterogeneity was expected across trials. Statistical heterogeneity was assessed using Cochran’s Q test and the I^2^ statistic. I^2^ values were interpreted as low, moderate, substantial, or considerable heterogeneity while also considering clinical differences between trials. A *p* value < 0.05 was considered statistically significant. Leave-one-out sensitivity analysis was performed for outcomes with notable heterogeneity. The main analysis was performed using brolucizumab 6 mg as an intervention group; however, a sensitivity analysis was performed by including 3 mg in the analysis. For studies with zero events in one treatment arm, the continuity-correction method implemented in the meta package was applied. Studies with zero events in both arms were not estimable and were excluded from the corresponding pooled effect estimate. All analyses were conducted in R version 4.5.3 (R Foundation for Statistical Computing, Vienna, Austria) using the meta and dmetar packages [22,23].

### 2.9. Assessment of Publication Bias

Small-study effects and possible publication bias were assessed visually using DOI plots when sufficient data were available [24]. These analyses were interpreted cautiously because fewer than 10 studies were available for each outcome.

## 3. Results

### 3.1. Selection Process

**Figure 1:**
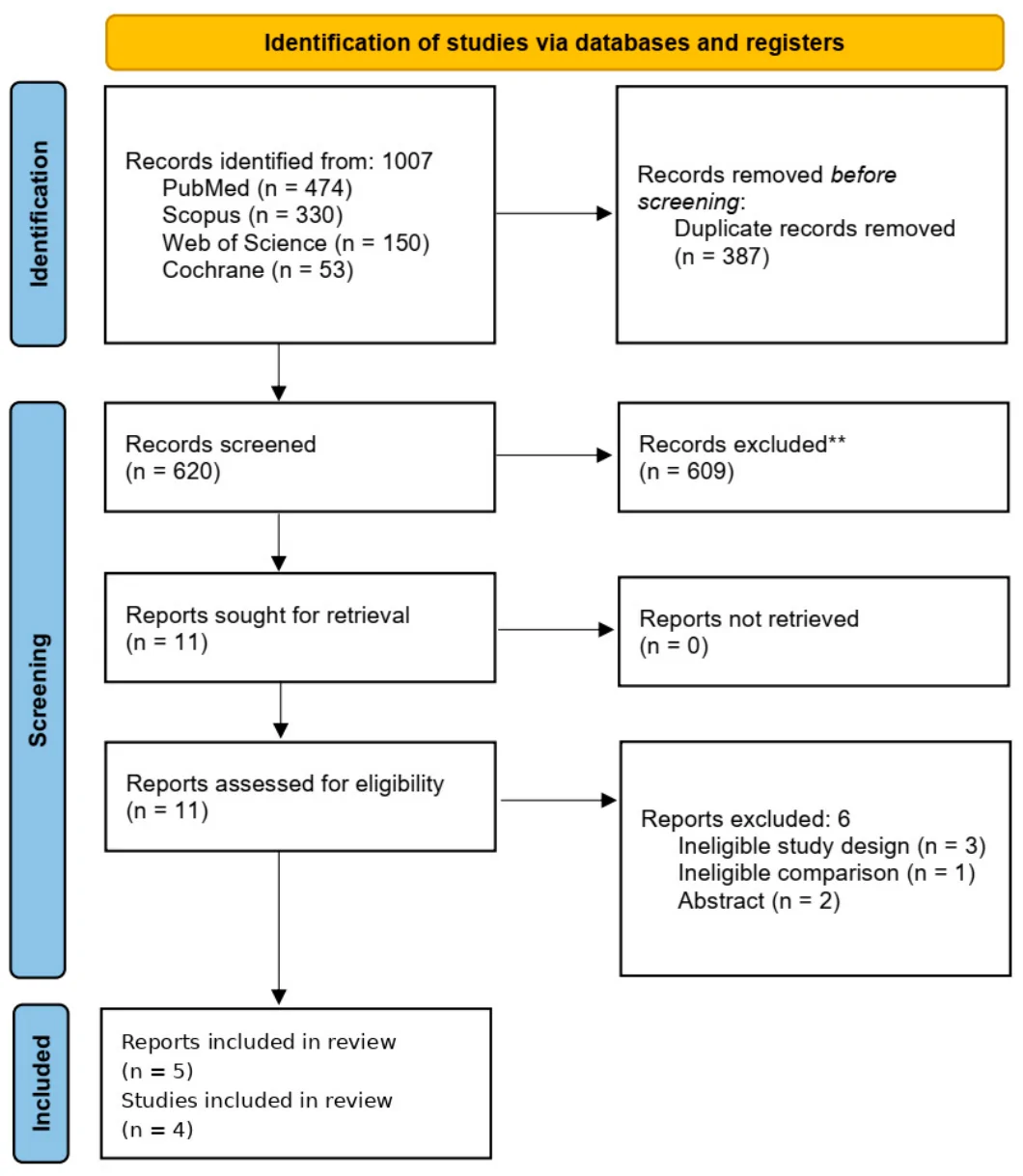
Preferred Reporting Items for Systematic Reviews and Meta-Analyses (PRISMA) flow diagram. ** Records excluded during title and abstract screening.

The literature search identified 1007 records, including 474 from PubMed, 330 from Scopus, 150 from Web of Science, and 53 from the Cochrane Library. After removal of 387 duplicates, 620 records were screened by title and abstract. Of these, 609 records were excluded, and 11 reports were assessed in full text. Six reports were excluded because of ineligible study design, ineligible comparison, abstract-only status, or lack of extractable data. Finally, five reports were included in the systematic review, representing four original RCTs [2,12,13,14,25]. The search and selection process is shown in the PRISMA flow diagram (Figure 1), and the completed PRISMA 2020 Checklist is provided as Supplementary Material.

### 3.2. Baseline Characteristics of the Included Studies and Patients

The review included four original phase 3 RCTs: KESTREL, KITE, KINGFISHER, and CONDOR. These trials enrolled 2132 participants, including 1251 patients in the brolucizumab groups and 881 patients in the control groups. Three trials evaluated patients with DME and used aflibercept as the comparator, whereas one trial evaluated patients with PDR and used PRP as the comparator. KESTREL included 566 patients, KITE included 360 patients, KINGFISHER included 517 patients, and CONDOR included 689 patients. Follow-up ranged from 52 to 100 weeks across the included reports. Further study-level details are summarized in Table 1.

Baseline characteristics were generally balanced between treatment groups within each trial. Mean age ranged from approximately 53 to 64 years across study arms. Baseline BCVA was lower in the diabetic macular edema trials than in the proliferative diabetic retinopathy trial, reflecting differences in enrolled populations. Baseline CST/CSFT was higher in the diabetic macular edema trials than in CONDOR. Most participants in the diabetic macular edema trials had type 2 diabetes, and intraretinal fluid was common at baseline. Detailed baseline characteristics are summarized in Table 2.

### 3.3. Quality Assessment

**Figure 2:**
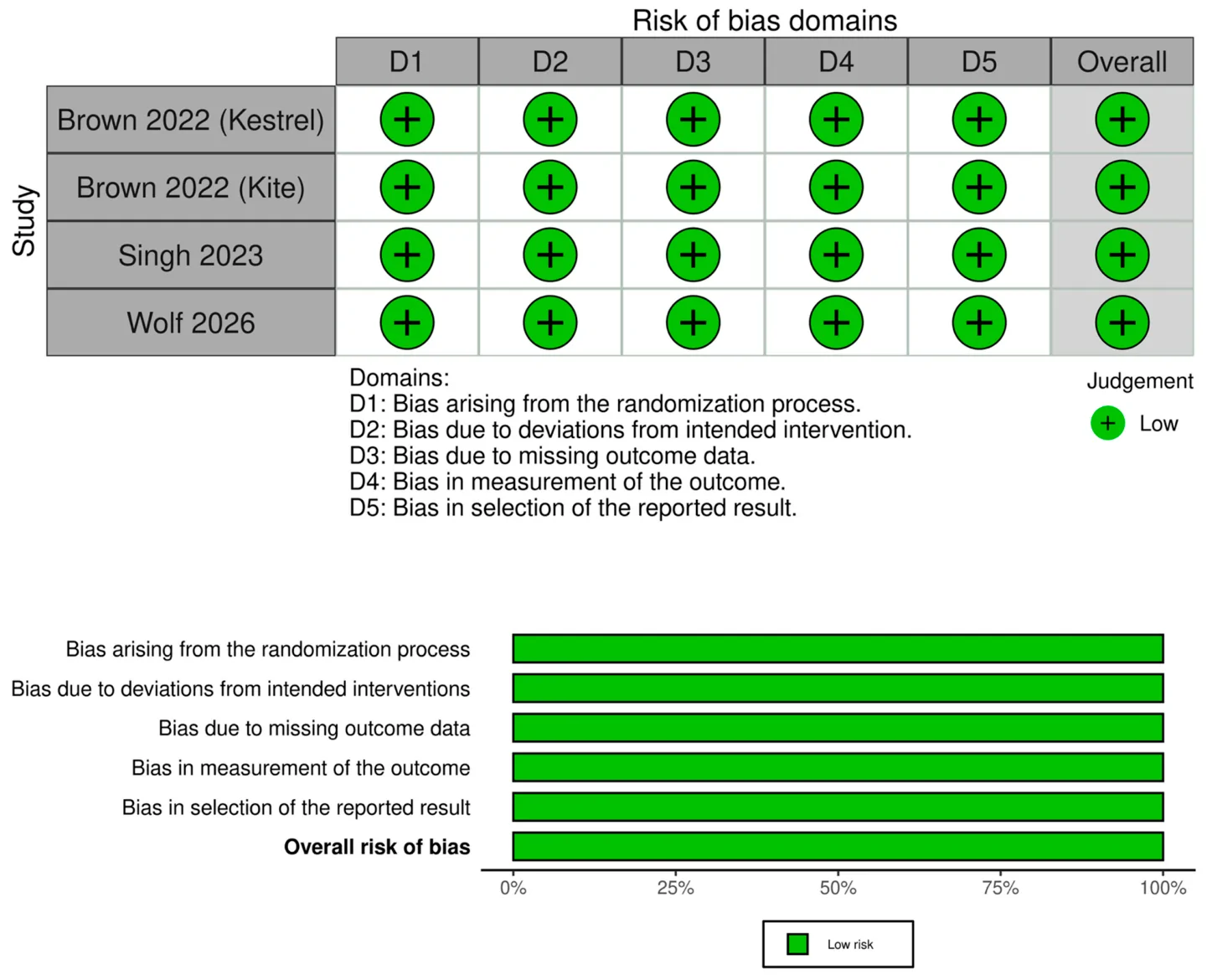
Cochrane Risk of Bias 2 (RoB 2) assessment for randomized controlled trials. Study labels correspond to Brown et al. [12], Singh et al. [14], and Wolf et al. [2].

All four included RCTs were judged to have low overall risk of bias. The included studies were rated as low risk across the main RoB 2 domains, including the randomization process, deviations from intended interventions, missing outcome data, outcome measurement, and selection of the reported result. The detailed risk-of-bias assessment is shown in Figure 2.

### 3.4. Outcomes

#### 3.4.1. Best-Corrected Visual Acuity

**Figure 3:**
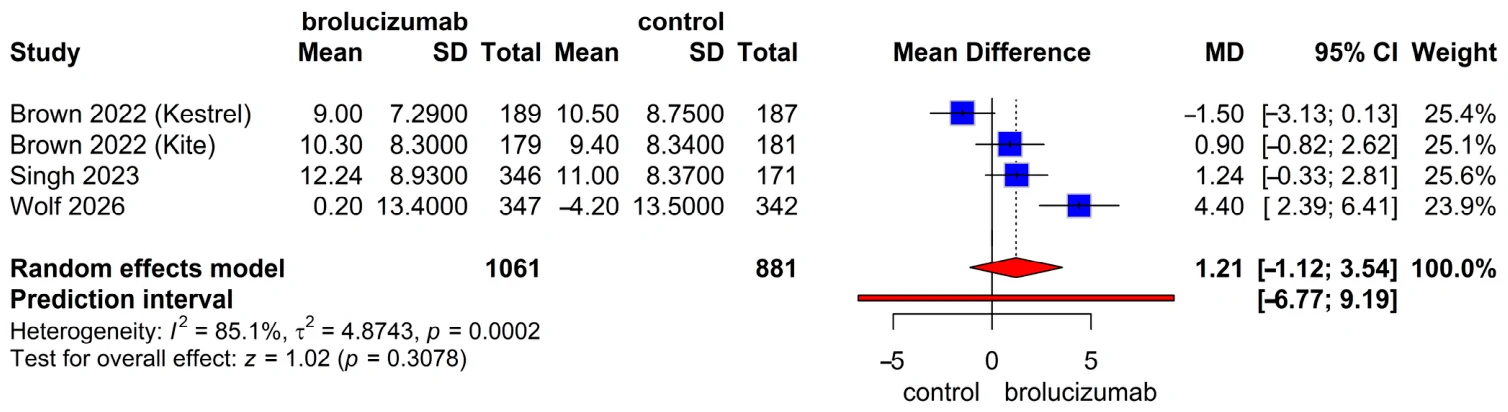
Forest plot for change in best-corrected visual acuity (BCVA). Study labels correspond to Brown et al. [12], Singh et al. [14], and Wolf et al. [2]. MD, mean difference; CI, confidence interval.

Four RCTs including 1942 patients reported change in best-corrected visual acuity. Brolucizumab was not associated with a statistically significant improvement in BCVA compared with control treatment. The pooled mean difference was 1.21 letters (95% CI −1.12 to 3.54; *p* = 0.3). Heterogeneity was considerable (I^2^ = 85.1%), indicating substantial variability across studies (Figure 3). Leave-one-out sensitivity analysis showed that the overall result remained generally non-significant after sequential omission of individual studies, although the magnitude and direction of effect varied across analyses, suggesting that the pooled estimate was influenced by between-study variability rather than a single dominant study (Supplementary Figure S1). Specifically, after excluding the CONDOR trial, which evaluated patients with PDR against PRP, the pooled estimate from the three DME trials remained non-significant (MD 0.21 letters, 95% CI −1.49 to 1.91), while heterogeneity decreased from 85.1% to 69%. This suggests that the absence of a statistically significant pooled BCVA benefit was not driven solely by the inclusion of CONDOR.

#### 3.4.2. Central Subfield Thickness

**Figure 4:**
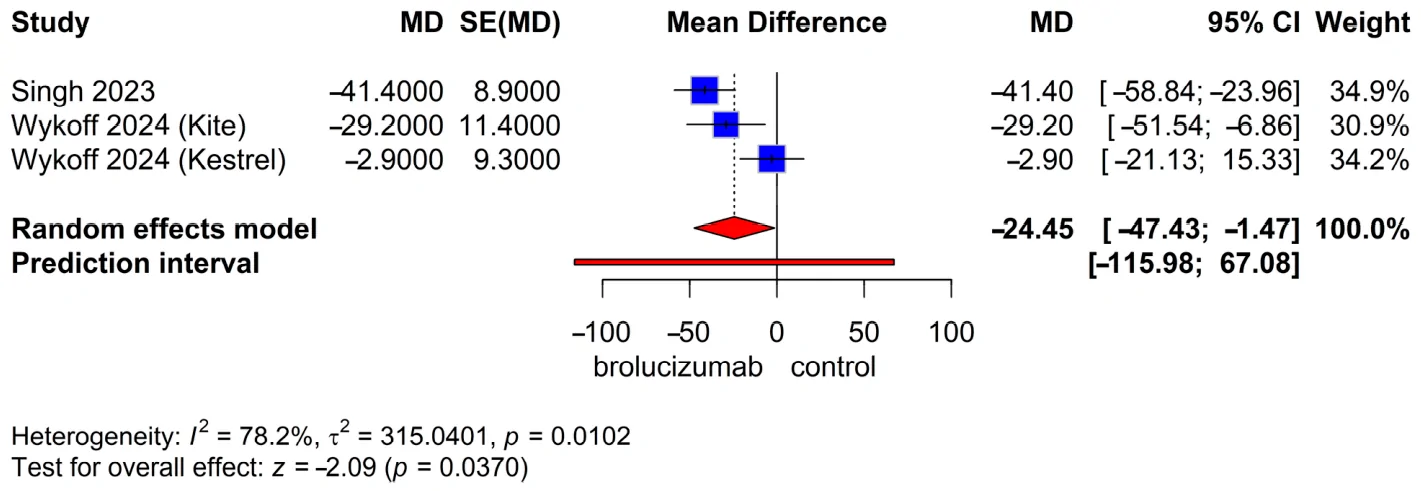
Forest plot for change in central subfield thickness (CST)/central subfield foveal thickness (CSFT). Study labels correspond to Wykoff et al. [13] and Singh et al. [14]. MD, mean difference; CI, confidence interval.

Three trial datasets contributed to the CST/CSFT analysis. Brolucizumab significantly reduced CST/CSFT compared with control treatment. The pooled mean difference was −24.45 µm (95% CI −47.43 to −1.47; *p* = 0.0370). Heterogeneity was substantial (I^2^ = 78.2%), suggesting important between-study variability in anatomical response (Figure 4). Leave-one-out sensitivity analysis showed that the direction of effect generally favored brolucizumab; however, statistical significance and heterogeneity were influenced by the omitted study, indicating that the anatomical finding should be interpreted with caution given the limited number of contributing datasets (Supplementary Figure S2).

#### 3.4.3. Ocular and Non-Ocular Adverse Events

**Figure 5:**
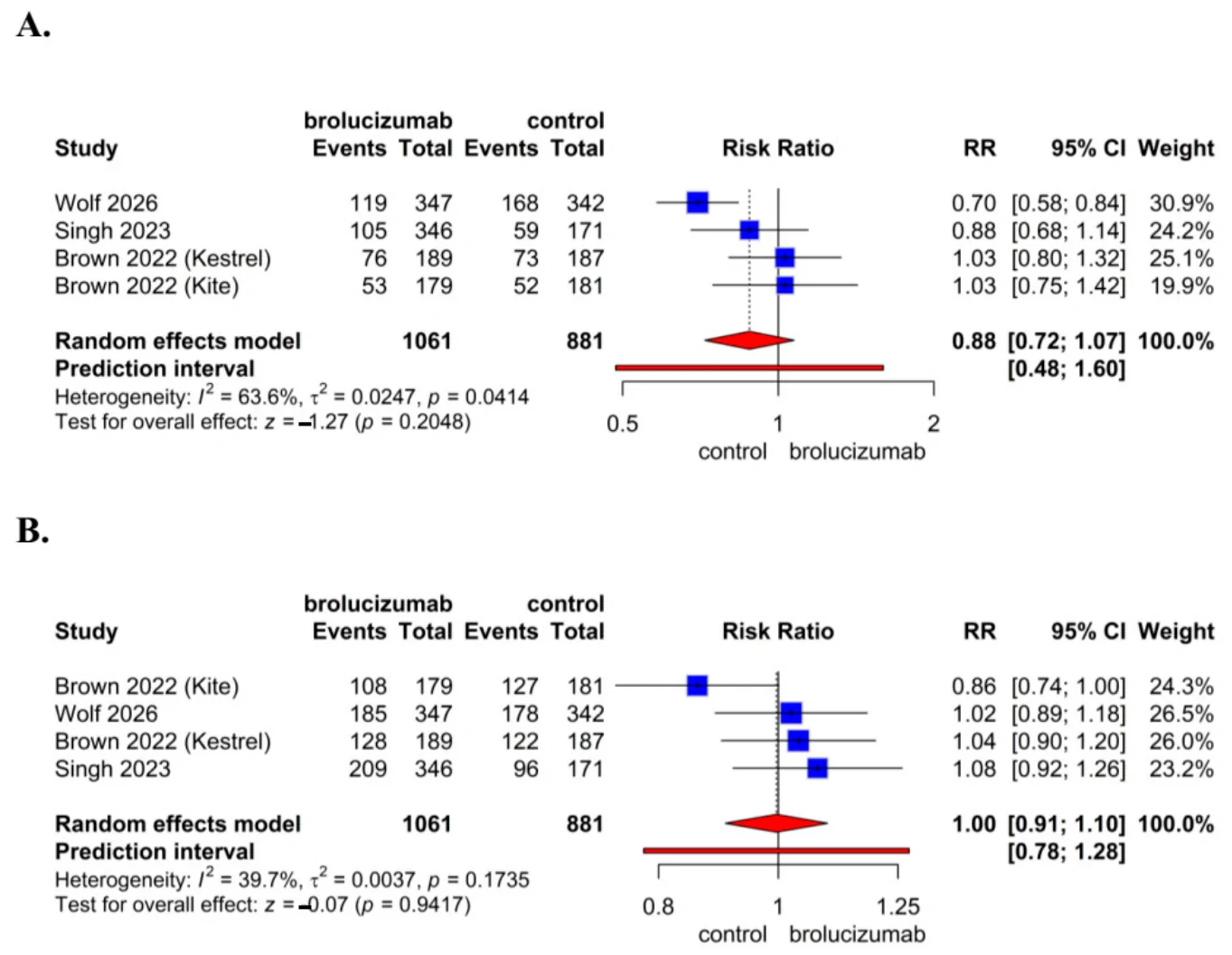
Forest plots for non-serious adverse events. (**A**) At least one ocular adverse event. (**B**) At least one non-ocular adverse event. Study labels correspond to Brown et al. [12], Singh et al. [14], and Wolf et al. [2]. RR, risk ratio; CI, confidence interval.

Four RCTs reported at least one ocular adverse event and at least one non-ocular adverse event. For at least one ocular adverse event, there was no statistically significant difference between brolucizumab 6 mg and control treatment (RR 0.88, 95% CI 0.72 to 1.07; *p* = 0.2), with substantial heterogeneity (I^2^ = 63.6%) (Figure 5A). Leave-one-out sensitivity analysis did not materially change the overall interpretation; however, heterogeneity decreased to 0% after exclusion of Wolf et al. [2] (CONDOR), indicating that this trial contributed substantially to the between-study heterogeneity (Supplementary Figure S3).

For at least one non-ocular adverse event, there was no statistically significant difference between groups (RR 1, 95% CI 0.91 to 1.10; *p* = 0.94), with low to moderate heterogeneity (I^2^ = 39.7%) (Figure 5B). Leave-one-out sensitivity analysis showed that the pooled estimates remained close to the null effect across sequential exclusions, supporting the stability of this finding (Supplementary Figure S4). Overall, these findings indicate no significant difference between brolucizumab and control treatment in the risk of common ocular or non-ocular adverse events (Figure 5).

#### 3.4.4. Serious Adverse Events

**Figure 6:**
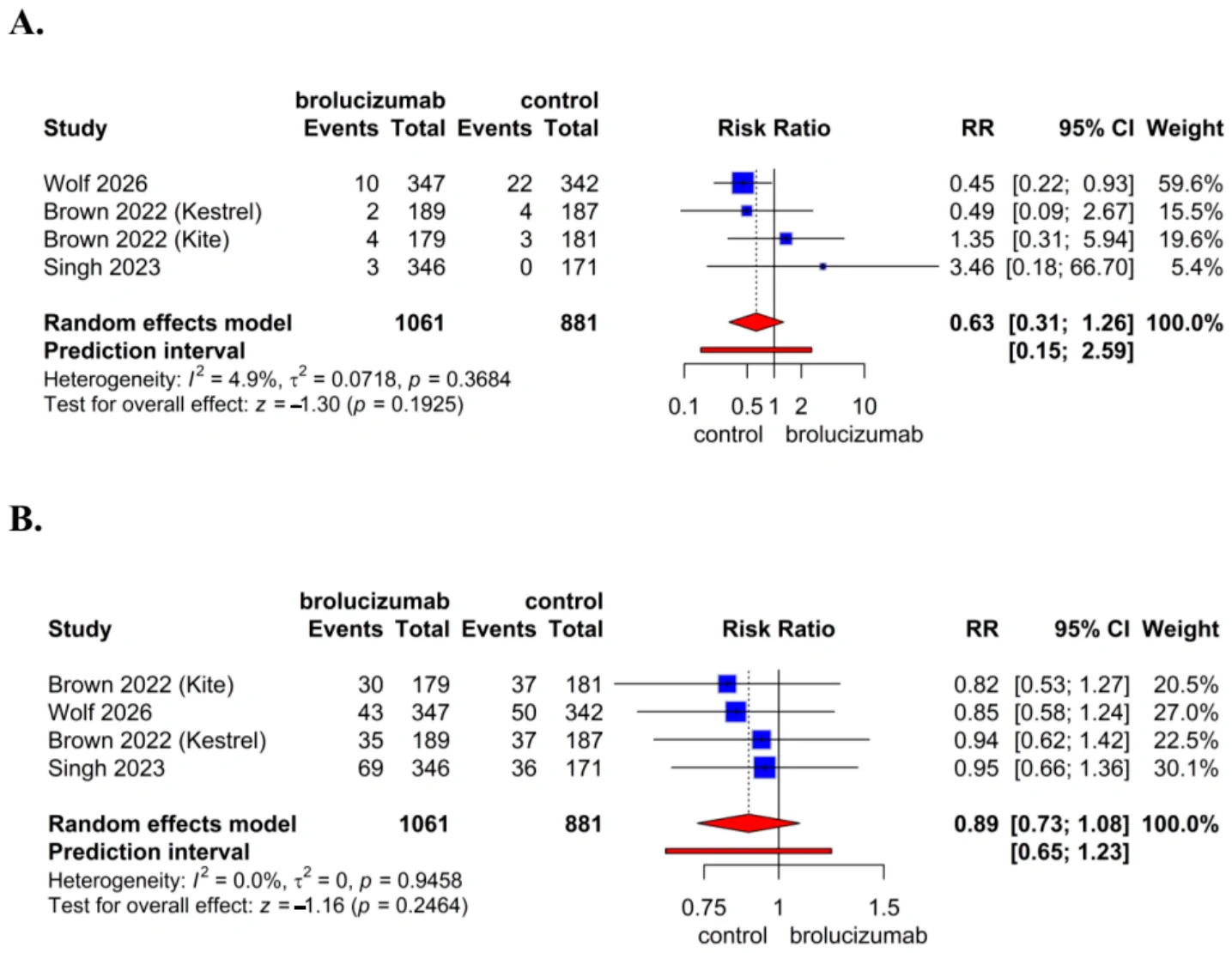
Forest plots for serious adverse events. (**A**) Serious ocular adverse events. (**B**) Serious non-ocular adverse events. Study labels correspond to Brown et al. [12], Singh et al. [14], and Wolf et al. [2]. RR, risk ratio; CI, confidence interval.

Four RCTs reported serious ocular and serious non-ocular adverse events. For serious ocular adverse events, no statistically significant difference was observed between brolucizumab and control treatment (RR 0.63, 95% CI 0.31 to 1.26; *p* = 0.19), with low heterogeneity (I^2^ = 4.9%) (Figure 6A). However, the CI was wide, reflecting the small number of events and limited precision. Leave-one-out sensitivity analysis showed that the overall interpretation remained limited by imprecision, with no clear evidence of a consistent difference between groups after sequential omission of individual studies (Supplementary Figure S5).

For serious non-ocular adverse events, brolucizumab was not associated with a statistically significant difference compared with control treatment (RR 0.89, 95% CI 0.73 to 1.08; *p* = 0.2464), with no observed heterogeneity (I^2^ = 0%) (Figure 6B). Although the point estimate favored brolucizumab, the CI crossed unity. Leave-one-out sensitivity analysis showed generally consistent estimates across sequential exclusions; however, the analysis remained limited by the number of trials and events (Supplementary Figure S6). Overall, no statistically significant difference in serious adverse-event rates was detected between groups, but the available evidence does not establish safety equivalence (Figure 6).

Specific ocular adverse events were uncommon, and none of the pooled estimates reached statistical significance. Intraocular inflammation was reported in 10 of 366 participants receiving brolucizumab and 4 of 368 controls across two trials. The pooled risk ratio was 2.35 (95% CI, 0.36–15.42; *p* = 0.3727), with moderate heterogeneity (I^2^ = 52.2%) (Supplementary Figure S7). Retinal vasculitis was reported in 2 of 535 participants receiving brolucizumab and none of 358 controls. The pooled risk ratio was 2.10 (95% CI, 0.22–20.10; *p* = 0.5199), with no observed heterogeneity (I^2^ = 0%) (Supplementary Figure S8).

For retinal vascular occlusion, events occurred in 5 of 714 participants receiving brolucizumab and 2 of 710 controls. The pooled estimate was not statistically significant (RR, 2.13; 95% CI, 0.46–9.87; *p* = 0.3341; I^2^ = 0%) (Supplementary Figure S9). Leave-one-out analysis yielded risk ratios ranging from 1.60 to 2.97, and all confidence intervals crossed unity, indicating that the interpretation was not driven by any single study (Supplementary Figure S10).

### 3.5. Sensitivity Analysis Based on Including Brolucizumab 3 mg

The sensitivity analysis based on the inclusion of brolucizumab 3 mg showed consistent results for the primary outcomes, indicating consistency across the two available doses (Supplementary Figures S11–S16).

### 3.6. Publication Bias Results

Publication bias was assessed using Doi plots and the Luis Furuya-Kanamori (LFK) index. Because each analysis included only three or four trial datasets, these findings should be interpreted cautiously. No important asymmetry was observed for BCVA (LFK index 0.63), CST/CSFT (0.61), ocular adverse events (1.22), or non-ocular adverse events (−0.71) (Supplementary Figures S17–S20). However, the Doi plot for serious ocular and non-ocular adverse events showed major asymmetry (LFK index 4.47 and −2.46, respectively) (Supplementary Figures S21 and S22). This may suggest possible small-study effects, although the result is uncertain because events were rare and the number of studies was small.

### 3.7. Narrative Synthesis of Brolucizumab 3 mg Versus 6 mg in KESTREL

In KESTREL, brolucizumab 6 mg appeared more favorable than 3 mg. The 6 mg dose met noninferiority versus aflibercept for BCVA at week 52, while the 3 mg dose was less consistent. Anatomically, more patients achieved CSFT < 280 µm with 6 mg than with 3 mg at week 52 (54.0% vs. 48.4%), and every-12-week (q12w) maintenance was also numerically higher with 6 mg (55.1% vs. 47.4%) [12]. Safety did not show a clear dose-related disadvantage for 6 mg. Ocular serious adverse events were reported in 1.1% with brolucizumab 6 mg versus 3.7% with brolucizumab 3 mg at week 52. Overall, KESTREL suggests that brolucizumab 6 mg provided more consistent efficacy and durability than 3 mg, without an obvious increase in ocular safety risk.

### 3.8. Certainty of Evidence

According to the GRADE assessment, the certainty of evidence was low for BCVA, low for CST/CSFT, moderate for at least one ocular adverse event, high for at least one non-ocular adverse event, low for serious ocular adverse events, and high for serious non-ocular adverse events. Downgrading was driven by inconsistency, indirectness, and imprecision. The full GRADE certainty assessment is presented in Table 3.

## 4. Discussion

In this systematic review and meta-analysis of four phase 3 RCTs, no statistically significant difference in BCVA was detected between brolucizumab and control treatment across the included DME and PDR trials. CST/CSFT was significantly reduced with brolucizumab in the three DME trial datasets. No statistically significant differences were detected in the assessed broad safety outcomes; however, these findings do not establish safety equivalence. Taken together, the results suggest an anatomical benefit in DME without demonstrated superiority in visual acuity, while safety conclusions remain limited by the small number of trials and rare events. In the separate analyses of brolucizumab-specific ocular events, the pooled point estimates were greater than unity for intraocular inflammation, retinal vasculitis, and retinal vascular occlusion. However, none reached statistical significance, and the very wide confidence intervals reflected the rarity of these events and the limited statistical power of the available RCTs. Therefore, these findings should not be interpreted as excluding a clinically important safety signal.

The neutral effect on BCVA is clinically important. In DME, anti-VEGF therapy improves vision by reducing vascular leakage and retinal edema, but visual recovery does not depend on retinal thickness alone [26,27]. It is also influenced by baseline BCVA, chronicity of edema, macular ischemia, photoreceptor integrity, and previous retinal damage [28,29]. This may explain why a measurable anatomical benefit did not translate into a significant pooled visual benefit. This interpretation is consistent with KINGFISHER, where brolucizumab was noninferior to aflibercept for BCVA, despite superior CST reduction and higher rates of fluid-free macula [14]. It is also consistent with KESTREL and KITE, where brolucizumab showed robust visual gains that were noninferior to aflibercept, while anatomical outcomes favored brolucizumab [12,13].

The lack of BCVA superiority may also reflect a ceiling effect, especially because the included studies were not fully homogeneous. The DME trials enrolled patients with lower baseline visual acuity and greater macular thickening, whereas CONDOR enrolled patients with PDR and relatively preserved baseline BCVA. In eyes with good starting vision, large gains in letters are less likely even when retinopathy improves. In CONDOR, brolucizumab preserved vision better than PRP, but the mean change was small because baseline BCVA was already high [2]. Therefore, the pooled visual estimate should not be interpreted as evidence that brolucizumab lacks clinical efficacy. Rather, it indicates that no statistically significant difference in mean BCVA change was detected across the included trials. Importantly, the result remained non-significant after exclusion of CONDOR, although heterogeneity was reduced, suggesting that the finding was not attributable solely to differences between the PDR and DME populations. Also, the BCVA-CST dissociation may reflect that retinal functional changes precede or even predict microvascular damage, indicating that the dysfunction of the neurovascular unit may be important in the development of DR [30].

The significant reduction in CST/CSFT is biologically plausible. Brolucizumab is a small single-chain antibody fragment with high affinity for VEGF-A, and its low molecular weight allows a high molar dose to be delivered intravitreally [31]. VEGF is a key driver of vascular permeability in DME, so stronger or more durable VEGF suppression may lead to better drying of the macula [32]. This explains why our CST/CSFT result favors brolucizumab and why individual trials repeatedly showed superior anatomical outcomes. KINGFISHER reported a greater CST reduction with brolucizumab than aflibercept at week 52 [14], and KESTREL/KITE showed fewer eyes with persistent intraretinal or subretinal fluid in the brolucizumab arms [12,13]. Despite that, the management strategy for KINGFISHER substantially differs from other trials, as they used the q4w fixed-dosing regimen, which can change the anatomical findings more than the other regimens in the other studies, which may explain why the results of the KINGFISHER trial are better than the other trials. Clinically, the anatomical benefit may matter even when BCVA does not immediately improve. Persistent macular fluid can indicate ongoing disease activity and may require more frequent monitoring or injections [33]. Previous evidence also suggests that a subset of DME patients continue to have persistent edema despite regular anti-VEGF therapy, which creates a need for agents with better drying ability or longer durability [34]. However, it is still uncertain whether better short-term drying with brolucizumab leads to better long-term visual outcomes. KINGFISHER itself noted that the relationship between optical coherence tomography (OCT) parameters and BCVA is incomplete, and superior anatomy did not translate into superior vision during 52 weeks [14].

Our findings agree with previous evidence syntheses. A focused network meta-analysis reported that brolucizumab 6 mg every 12 or 8 weeks was generally comparable or superior to aflibercept and ranibizumab regimens for visual and anatomical outcomes, with a generally favorable benefit-risk profile except for ocular inflammatory and occlusive events [35]. A later RCT meta-analysis comparing brolucizumab with aflibercept in DME found no significant difference in BCVA but reported significantly greater CST improvement with brolucizumab [36]. Our analysis extends this literature by incorporating the newer CONDOR PDR trial and the recent post hoc evidence from KINGFISHER, allowing a broader assessment across diabetic eye disease rather than DME alone [2,25]. Despite the inclusion of the new RCT compared to the previous meta-analysis, the findings are still the same, indicating consistent findings across the RCTs.

The safety findings require balanced interpretation. Overall ocular adverse events were not significantly increased with brolucizumab in our pooled analysis. This agrees with the recent DME meta-analysis, which also found no statistically significant difference in ocular adverse events between brolucizumab and aflibercept [35]. However, brolucizumab has a known safety concern related to intraocular inflammation, retinal vasculitis, and retinal vascular occlusion [37]. The National Institute for Health and Care Excellence (NICE) specifically noted concern about intraocular inflammation with brolucizumab based on postmarketing experience in neovascular age-related macular degeneration, although it considered the event uncommon in DME [38,39]. This distinction is important. Our pooled adverse-event outcomes summarize broad categories, not only inflammation-related events. The wide confidence intervals around the pooled safety estimates, particularly for serious ocular adverse events, indicate substantial imprecision and limited statistical power rather than confirmation of comparable safety. In CONDOR, intraocular inflammation including retinal vasculitis was more frequent with brolucizumab than PRP, although overall ocular adverse events were more frequent in the PRP arm [2]. In KINGFISHER, rates of intraocular inflammation and retinal vasculitis were low, and no new safety signal was identified [14]. The available trial-level evidence did not detect statistically significant differences in the assessed broad safety outcomes. However, these findings should not be interpreted as evidence of safety equivalence, particularly because the analyses included only four randomized controlled trials and a limited number of rare serious ocular events. Clinicians should still monitor carefully for inflammatory or occlusive events.

No statistically significant differences were detected in non-ocular adverse events or serious non-ocular adverse events. This is clinically relevant because patients with DME and DR often have systemic vascular comorbidities. Anti-VEGF agents are given locally, but systemic safety remains important in patients with diabetes, cardiovascular disease, or previous vascular events. However, the available trials do not establish systemic safety equivalence or exclude modest or uncommon systemic risks.

The PDR data should be interpreted separately from the DME data. PRP has long been the standard treatment for PDR, but it can be associated with visual field loss, night vision problems, macular edema, and other laser-related complications. Anti-VEGF therapy has emerged as an alternative or adjunct to PRP in selected patients. In CONDOR, brolucizumab was noninferior and superior to PRP for BCVA change at week 54 and produced a higher proportion of patients with no PDR at week 54 [2]. These findings support the potential role of brolucizumab in PDR, but the evidence is still based on one trial and should not be generalized to all PDR settings without considering follow-up adherence, cost, access and long-term durability.

### 4.1. Clinical Implications

Current guidelines already support anti-VEGF therapy as a central treatment option for DME with visual impairment. NICE recommends anti-VEGF therapy for center-involving DME with visual impairment when central retinal thickness is 400 µm or more, and recommends considering anti-VEGF therapy or macular laser when central retinal thickness is below 400 µm [38]. NICE also lists ranibizumab, brolucizumab, faricimab, and aflibercept as licensed anti-VEGF options for visual impairment due to DME. For brolucizumab specifically, NICE concluded that evidence from KESTREL and KITE suggested similar clinical effectiveness to aflibercept and likely similar effectiveness to ranibizumab. Our findings support this guideline position. Brolucizumab appears to be a reasonable anti-VEGF option for DME when the clinical goal is to maintain visual outcomes while improving anatomical drying. The observed CST/CSFT reduction may be useful in eyes with persistent fluid or high treatment burden. However, our results do not show clear visual superiority, so brolucizumab should not be presented as universally better than aflibercept or other anti-VEGF agents. A neutral interpretation is that it may offer anatomical advantages without a statistically significant difference in visual efficacy.

For PDR, guidelines are more cautious. NICE recommends anti-VEGF treatment for PDR that remains active after complete PRP and as a temporary treatment when vitreous hemorrhage or cataract prevents PRP [40]. It also noted that, in August 2024, ranibizumab was the only anti-VEGF licensed for PDR in that setting, with other anti-VEGF use considered off-label. American Academy of Ophthalmology (AAO) guidance also recognizes anti-VEGF therapy as effective for PDR while maintaining an important role for PRP [7]. Our findings may add evidence that brolucizumab can be an effective alternative to PRP in selected PDR patients, but the evidence is not yet strong enough to change practice broadly without longer follow-up and additional comparative studies.

### 4.2. Strengths, Limitations, and Recommendations

The main strength of this review is its focus on randomized evidence. We included four phase 3 RCTs and separated visual, anatomical, ocular safety, and systemic safety outcomes. Another strength is the inclusion of newer evidence, especially KINGFISHER and CONDOR, which allows a more updated synthesis than earlier meta-analyses limited mainly to KESTREL and KITE. Several limitations should be considered. First, the number of included trials was small, which limits precision and makes publication-bias assessment unreliable. Second, there was clinical heterogeneity because most trials studied DME with aflibercept as the comparator, while CONDOR studied PDR with PRP as the comparator. Third, follow-up duration and dosing schedules differed between trials. KINGFISHER used every 4-week brolucizumab dosing, which is not the approved maintenance regimen for DME. Fourth, rare inflammatory and occlusive events may be underpowered in RCT-level meta-analysis. These methodological limitations may bias the study findings, so the results should be interpreted with caution, and further RCTs in DME are still needed to determine the impact of brolucizumab on these patients. Future trials should compare brolucizumab with current anti-VEGF options using approved dosing regimens and should report standardized inflammatory and vascular occlusive safety outcomes. More PDR trials are needed, especially against PRP and other anti-VEGF agents, with longer follow-up and careful assessment of adherence, rescue treatment, recurrent neovascularization, vitreous hemorrhage, and tractional complications.

## 5. Conclusions

Across the four included trials, brolucizumab was not associated with a statistically significant difference in BCVA. In the three DME trials, brolucizumab was associated with a significantly greater reduction in CST/CSFT. No statistically significant differences were detected in the assessed broad safety outcomes; however, the limited trial-level evidence does not establish safety equivalence or exclude rare serious inflammatory and vascular occlusive events. Brolucizumab may be useful for selected patients with DME when anatomical drying or durability is prioritized, whereas its role in PDR requires longer-term evidence. These findings should be interpreted cautiously because the included studies differed in population, comparator, dosing schedule, and follow-up.

## Figures and Tables

**Table 1:** Summary of included studies.

Study ID	Study Design	Country	Study Duration	Follow-Up	Inclusion Criteria	Overall Sample Size	Intervention Group	Control Group
Brown [12] (KESTREL)	Phase 3 RCT	Multicenter	30 July 2018 and 14 November 2019	100 weeks; published analysis at week 52 in Brown 2022 [12] and at week 100 in Wykoff 2024 [13]	patients aged ≥ 18 with type 1 or 2 diabetes with HbA1c < 10%, macular edema and BCVA score between 78 and 23 letters	566	Name: brolucizumab Dose: 3 and 6 mg Route: intravitreal Duration: 24 weeks Sample size: 379	Name: aflibercept Dose: 2 mg Route: intravitreal Duration: 24 weeks Sample size: 187
Brown [12] (KITE)	Phase 3 RCT	Multicenter	10 August 2018 and 2 July 2019	100 weeks; published analysis at week 52 in Brown 2022 [12] and at week 100 in Wykoff 2024 [13]	patients aged ≥ 18 with type 1 or 2 diabetes with HbA1c < 10%, macular edema and BCVA score between 78 and 23 letters	360	Name: brolucizumab Dose: 6 mg Route: intravitreal Duration: 24 weeks Sample size: 179	Name: aflibercept Dose: 2 mg Route: intravitreal Duration: 24 weeks Sample size: 181
Singh [14] (KINGFISHER)	Phase 3 RCT	Multicenter	5 September 2019, and 11 March 2020	52 weeks	patients aged ≥ 18 with type 1 or 2 diabetes with HbA1c < 12%, macular edema	517	Name: brolucizumab Dose: 6 mg Route: intravitreal Duration: every 4 weeks Sample size: 346	Name: aflibercept Dose: 2 mg Route: intravitreal Duration: every 4 weeks Sample size: 171
Wolf [2] (CONDOR)	Phase 3 RCT	Multicenter	NR	96-week trial; published analysis at week 54	patients aged ≥ 18 with type 1 or 2 diabetes with HbA1c < 12%, PDR	689	Name: brolucizumab Dose: 6 mg Route: intravitreal Duration: Three loading injections every 6 weeks, followed by injections every 12 weeks; from week 48, the treatment interval could be extended by 6-week increments up to every 24 weeks. Sample size: 347	Name: PRP Route: Panretinal photocoagulation Duration: Delivered in 1 to 4 treatment sessions up to week 12, followed by additional panretinal photocoagulation treatment as needed. Sample size: 342

**Abbreviations:** BCVA, best-corrected visual acuity; DME, diabetic macular edema; HbA1c, glycated hemoglobin; NR, not reported; PDR, proliferative diabetic retinopathy; PRP, panretinal photocoagulation; RCT, randomized controlled trial; VEGF, vascular endothelial growth factor.

**Table 2:** Baseline characteristics of included studies.

Study ID	Arm	Age, Years Mean (SD)	Male Gender N (%)	BCVA Mean (SD)	CST Mean (SD)	Type 2 Diabetes N (%)	HbA1c Mean (SD)	Intraretinal Fluid N (%)	Subretinal Fluid N (%)
Brown [12] (KESTREL)	brolucizumab (3 mg)	64.4 (9.76)	119 (62.6%)	65.7 (11.09)	456 (118)	180 (94.7%)	7.52 (1.16)	190 (100.0%)	60 (31.6%)
	brolucizumab (6 mg)	62.4 (10.14)	110 (58.2%)	66.6 (9.67)	453 (123)	177 (93.7%)	7.69 (1.07)	189 (100.0%)	62 (32.8%)
	aflibercept	63.9 (10.09)	126 (67.4%)	65.2 (12.38)	476 (136)	181 (96.8%)	7.44 (1.13)	184 (98.4%)	61 (32.6%)
Brown [12] (KITE)	brolucizumab	62.3 (10.55)	120 (67.0%)	66 (10.77)	481 (132)	160 (89.4%)	7.55 (1.17)	176 (98.3%)	56 (31.3%)
	aflibercept	62.2 (9.48)	115 (63.5%)	63.7 (11.7)	484 (135)	174 (96.1%)	7.46 (1.16)	179 (98.9%)	67 (37.0%)
Singh [14] (KINGFISHER)	brolucizumab	60.9 (10.59)	194 (56.1%)	61.3 (10.14)	514.1 (138.94)	327 (94.5%)	7.84 (1.48)	344 (99.4%)	128 (37.0%)
	aflibercept	60.2 (9.31)	105 (61.4%)	60.5 (11.27)	511.2 (156.29)	162 (94.7%)	7.98 (1.58)	170 (99.4%)	59 (34.5%)
Wolf [2] (CONDOR)	brolucizumab	53.2 (11.88)	203 (58.5%)	77.2 (10.23)	274.3 (48.55)	NR	NR	103 (29.7%)	5 (1.4%)
	PRP	54.7 (10.83)	210 (61.4%)	77 (10.87)	276.7 (39.3)	NR	NR	121 (35.5%)	6 (1.8%)

**Abbreviations:** BCVA, best-corrected visual acuity; CST, central subfield thickness; HbA1c, glycated hemoglobin; N, number of participants; NR, not reported; PRP, panretinal photocoagulation; SD, standard deviation.

**Table 3:** GRADE certainty of evidence.

Certainty Assessment							No. of Patients		Effect		Overall Certainty
No. of Studies	Study Design	Risk of Bias	Inconsistency	Indirectness	Imprecision	Other Considerations	Brolucizumab	Control Treatment	Relative (95% CI)	Absolute (95% CI)	
BCVA											
4	randomized trials	not serious	serious ^a^	serious ^b^	not serious	none	1061	881	-	MD 1.21 letters higher (1.12 lower to 3.54 higher)	⨁⨁◯◯ Low ^a,b^
CST/CSFT											
3	randomized trials	not serious	serious ^a^	not serious	serious ^c^	none	714	539	-	MD 24.45 µm lower (47.43 lower to 1.47 lower)	⨁⨁◯◯ Low ^a,c^
At least one ocular adverse event											
4	randomized trials	not serious	serious ^a^	not serious	not serious	none	353/1061 (33.3%)	352/881 (40.0%)	RR 0.88 (0.72 to 1.07)	48 fewer per 1000 (from 112 fewer to 28 more)	⨁⨁⨁◯ Moderate ^a^
At least one non-ocular adverse event											
4	randomized trials	not serious	not serious	not serious	not serious	none	630/1061 (59.4%)	523/881 (59.4%)	RR 1.00 (0.91 to 1.10)	0 fewer per 1000 (from 53 fewer to 59 more)	⨁⨁⨁⨁ High
At least one serious ocular adverse event											
4	randomized trials	not serious	not serious	not serious	very serious ^d^	none	19/1061 (1.8%)	29/881 (3.3%)	RR 0.63 (0.31 to 1.26)	12 fewer per 1000 (from 23 fewer to 9 more)	⨁⨁◯◯ Low ^d^
At least one serious non-ocular adverse event											
4	randomized trials	not serious	not serious	not serious	not serious	none	177/1061 (16.7%)	160/881 (18.2%)	RR 0.89 (0.73 to 1.08)	20 fewer per 1000 (from 49 fewer to 15 more)	⨁⨁⨁⨁ High

CI: confidence interval; MD: mean difference; RR: risk ratio. Explanations: ^a^. Serious inconsistency due to substantial or considerable heterogeneity. ^b^. Serious indirectness due to pooling clinically different DME and PDR populations and comparators. ^c^. Serious imprecision because the confidence interval was close to the null and statistical significance was sensitive to leave-one-out analysis. ^d^. Very serious imprecision due to the small number of rare events and the wide confidence interval. GRADE certainty ratings: ⨁⨁⨁⨁ = high; ⨁⨁⨁◯ = moderate; ⨁⨁◯◯ = low; and ⨁◯◯◯ = very low.

## Data Availability

All data are provided in the manuscript and its related Supplementary Materials.

## Supplementary Materials

The following supporting information can be downloaded at https://www.mdpi.com/article/10.3390/jcm15176518/s1. Table S1: Search strategy; Figure S1: Leave-one-out sensitivity analysis for best-corrected visual acuity; Figure S2: Leave-one-out sensitivity analysis for central subfield thickness/central subfield foveal thickness; Figure S3: Leave-one-out sensitivity analysis for at least one ocular adverse event; Figure S4: Leave-one-out sensitivity analysis for at least one non-ocular adverse event; Figure S5: Leave-one-out sensitivity analysis for serious ocular adverse events; Figure S6: Leave-one-out sensitivity analysis for serious non-ocular adverse events; Figure S7: Forest plot comparing intraocular inflammation between brolucizumab and control groups; Figure S8: Forest plot comparing retinal vasculitis between brolucizumab and control groups; Figure S9: Forest plot comparing retinal vascular occlusion between brolucizumab and control groups; Figure S10: Leave-one-out sensitivity analysis for retinal vascular occlusion; Figure S11: Sensitivity analysis for best-corrected visual acuity by including the brolucizumab 3 mg dose in the analysis; Figure S12: Sensitivity analysis for central subfield thickness/central subfield foveal thickness by including the brolucizumab 3 mg dose in the analysis; Figure S13: Sensitivity analysis for at least one ocular adverse event by including the brolucizumab 3 mg dose in the analysis; Figure S14: Sensitivity analysis for at least one non-ocular adverse event by including the brolucizumab 3 mg dose in the analysis; Figure S15: Sensitivity analysis for serious ocular adverse events by including the brolucizumab 3 mg dose in the analysis; Figure S16: Sensitivity analysis for serious non-ocular adverse events by including the brolucizumab 3 mg dose in the analysis; Figure S17: Doi plot for publication bias assessment of best-corrected visual acuity; Figure S18: Doi plot for publication bias assessment of central subfield thickness/central subfield foveal thickness; Figure S19: Doi plot for publication bias assessment of at least one ocular adverse event; Figure S20: Doi plot for publication bias assessment of at least one non-ocular adverse event; Figure S21: Doi plot for publication bias assessment of serious non-ocular adverse events; Figure S22: Doi plot for publication bias assessment of serious ocular adverse events; PRISMA 2020 Checklist.

## Abbreviations

DM, diabetes mellitus; DR, diabetic retinopathy; DME, diabetic macular edema; PDR, proliferative diabetic retinopathy; PRP, panretinal photocoagulation; anti-VEGF, anti-vascular endothelial growth factor; VEGF, vascular endothelial growth factor; RCT, randomized controlled trial; PRISMA, Preferred Reporting Items for Systematic Reviews and Meta-Analyses; PICO, Population, Intervention, Comparator, Outcome; PROSPERO, International Prospective Register of Systematic Reviews; BCVA, best-corrected visual acuity; CST, central subfield thickness; CSFT, central subfield foveal thickness; RoB 2, Cochrane Risk of Bias 2 tool; GRADE, Grading of Recommendations Assessment, Development and Evaluation; MD, mean difference; CI, confidence interval; RR, risk ratio; I^2^, I-squared statistic; LFK index, Luis Furuya-Kanamori index; NICE, National Institute for Health and Care Excellence; AAO, American Academy of Ophthalmology.
