# Supplementary material for: Safety and Efficacy of Brolucizumab in the Treatment of Patients with Diabetic Macular Edema and Diabetic Retinopathy: A Systematic Review and Meta-Analysis
Source: J Clin Med. 2026 Aug 23;15(17):6518. doi: 10.3390/jcm15176518 (PMC13565801; doi:10.3390/jcm15176518)
Supplement: Supplementary file 1 [file jcm-15-06518-s001.zip › jcm-4397118-supplementary.pdf]

Supplementary Materials

Supplementary Table S1: Search strategy

Supplementary Figure S1: Leave-one-out sensitivity analysis for best-corrected visual acuity.

Supplementary Figure S2: Leave-one-out sensitivity analysis for central subfield thickness/central subfield foveal thickness.

Supplementary Figure S3: Leave-one-out sensitivity analysis for at least one ocular adverse event.

Supplementary Figure S4: Leave-one-out sensitivity analysis for at least one non-ocular adverse event.

Supplementary Figure S5: Leave-one-out sensitivity analysis for serious ocular adverse events.

Supplementary Figure S6: Leave-one-out sensitivity analysis for serious non-ocular adverse events.

Supplementary Figure S7: Forest plot comparing intraocular inflammation between brolucizumab and control groups.

Supplementary Figure S8: Forest plot comparing retinal vasculitis between brolucizumab and control groups.

Supplementary Figure S9: Forest plot comparing retinal vascular occlusion between brolucizumab and control groups.

Supplementary Figure S10: Leave-one-out sensitivity analysis for retinal vascular occlusion.

Supplementary Figure S11: Sensitivity analysis for best-corrected visual acuity by including brolucizumab 3mg dose in the analysis

Supplementary Figure S12: Sensitivity analysis for central subfield thickness/central subfield foveal thickness by including brolucizumab 3mg dose in the analysis

Supplementary Figure S13: Sensitivity analysis for at least one ocular adverse event by including brolucizumab 3mg dose in the analysis

Supplementary Figure S14: Sensitivity analysis for at least one non-ocular adverse event by including brolucizumab 3mg dose in the analysis

Supplementary Figure S15: Sensitivity analysis for serious ocular adverse events by including brolucizumab 3mg dose in the analysis

Supplementary Figure S16: Sensitivity analysis for serious non-ocular adverse events by including brolucizumab 3mg dose in the analysis

Supplementary Figure S17: Doi plot for publication bias assessment of best-corrected visual acuity.

Supplementary Figure S18: Doi plot for publication bias assessment of central subfield thickness/central subfield foveal thickness.

Supplementary Figure S19: Doi plot for publication bias assessment of at least one ocular adverse event.

Supplementary Figure S20: Doi plot for publication bias assessment of at least one non-ocular adverse event.

Supplementary Figure S21: Doi plot for publication bias assessment of serious non-ocular adverse events.

Supplementary Figure S22: Doi plot for publication bias assessment of serious ocular adverse events.

PRISMA Checklist

Database	Search Strategy
PubMed	(Brolucizumab OR Beovu OR "brolucizumab" [Supplementary Concept])<br>AND<br>(diabetic retinopathy OR Diabetic Retinopathies OR diabetic macular edema OR macular edema OR Cystoid Macular Dystrophy OR Cystoid Macular Edema OR Central Retinal Edema OR Irvine Gass Syndrome OR Irvine-Gass Syndrome OR "Diabetic Retinopathy"[Mesh] OR "Macular Edema"[Mesh] OR maculopathy OR Macular Dystrophy)
Scopus	(Brolucizumab OR Beovu)<br>AND<br>("diabetic retinopathy" OR "Diabetic Retinopathies" OR "diabetic macular edema" OR "macular edema" OR "Cystoid Macular Dystrophy" OR "Cystoid Macular Edema" OR "Central Retinal Edema" OR "Irvine Gass Syndrome" OR "Irvine-Gass Syndrome" OR maculopathy OR "Macular Dystrophy")
Web of Science	(Brolucizumab OR Beovu)<br>AND<br>(diabetic retinopathy OR Diabetic Retinopathies OR diabetic macular edema OR macular edema OR Cystoid Macular Dystrophy OR Cystoid Macular Edema OR Central Retinal Edema OR Irvine Gass Syndrome OR Irvine-Gass Syndrome OR maculopathy OR Macular Dystrophy)
Cochrane Library	(Brolucizumab OR Beovu)<br>AND<br>(diabetic retinopathy OR Diabetic Retinopathies OR diabetic macular edema OR macular edema OR Cystoid Macular Dystrophy OR Cystoid Macular Edema OR Central Retinal Edema OR Irvine Gass Syndrome OR Irvine-Gass Syndrome OR maculopathy OR Macular Dystrophy)

Supplementary Table S1. Search strategy

Supplementary Figure S1. Leave-one-out sensitivity analysis for best-corrected visual acuity.

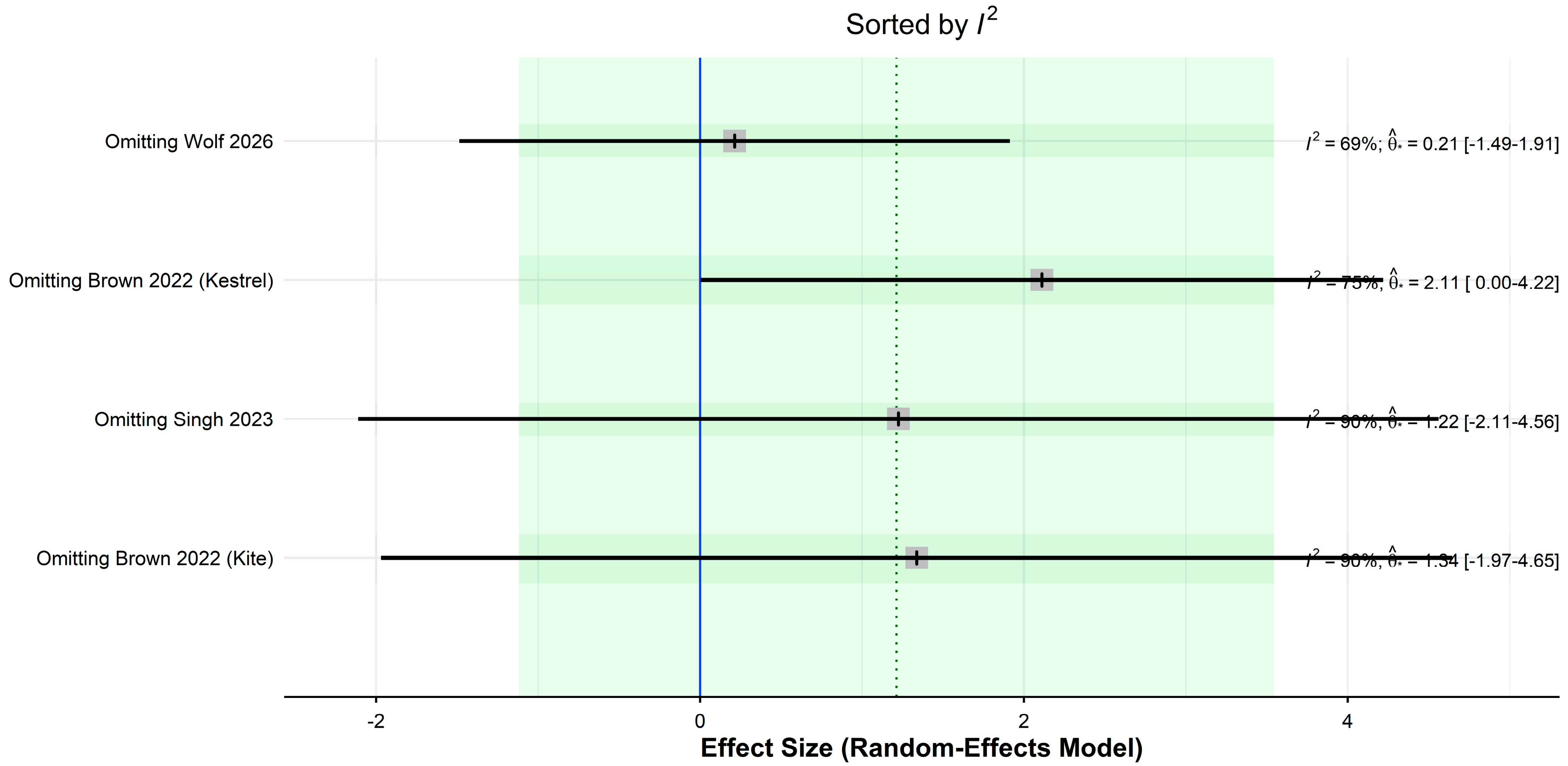

Supplementary Figure S2. Leave-one-out sensitivity analysis for central subfield thickness/central subfield foveal thickness.

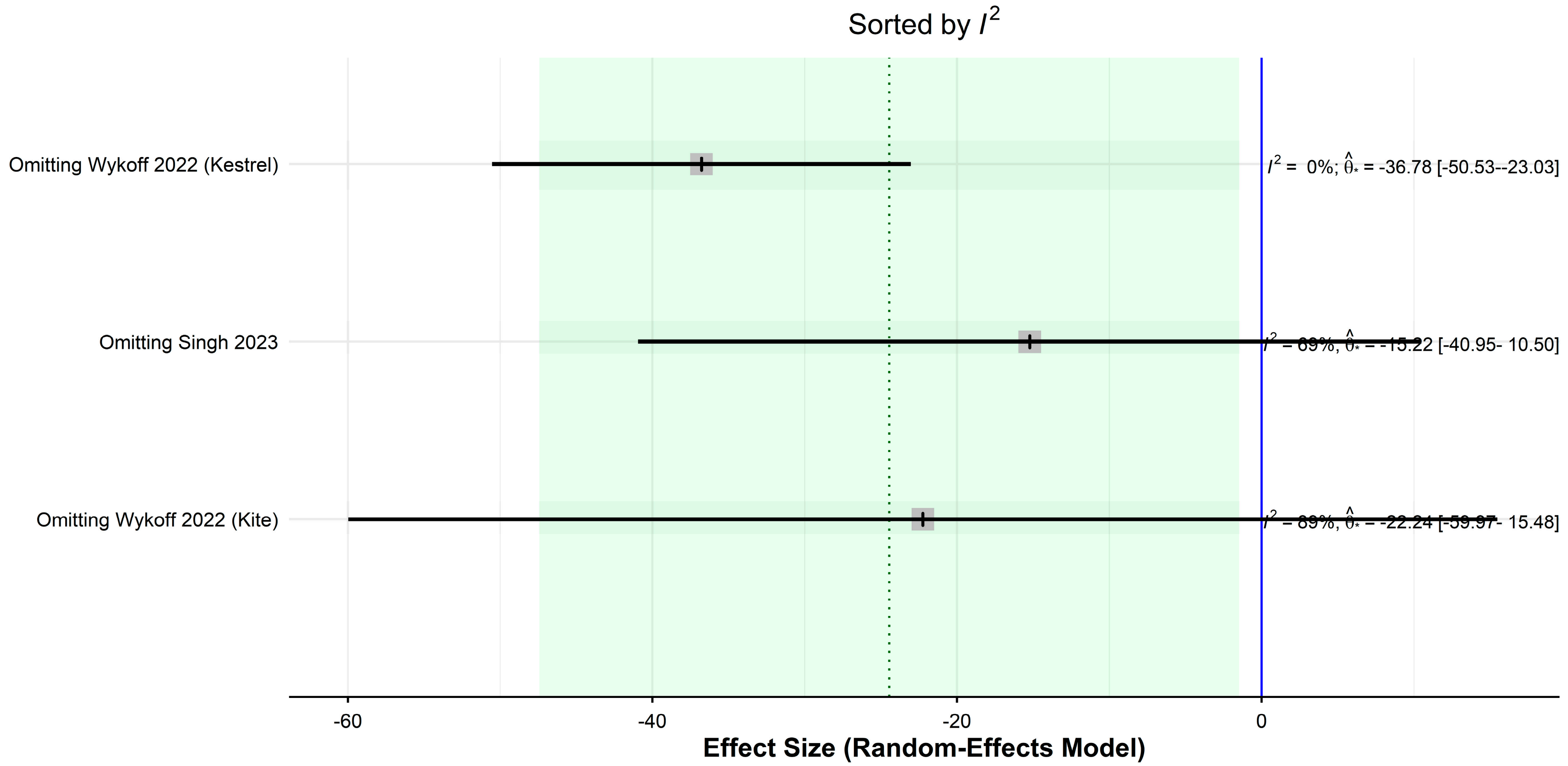

Supplementary Figure S3. Leave-one-out sensitivity analysis for at least one ocular adverse event.

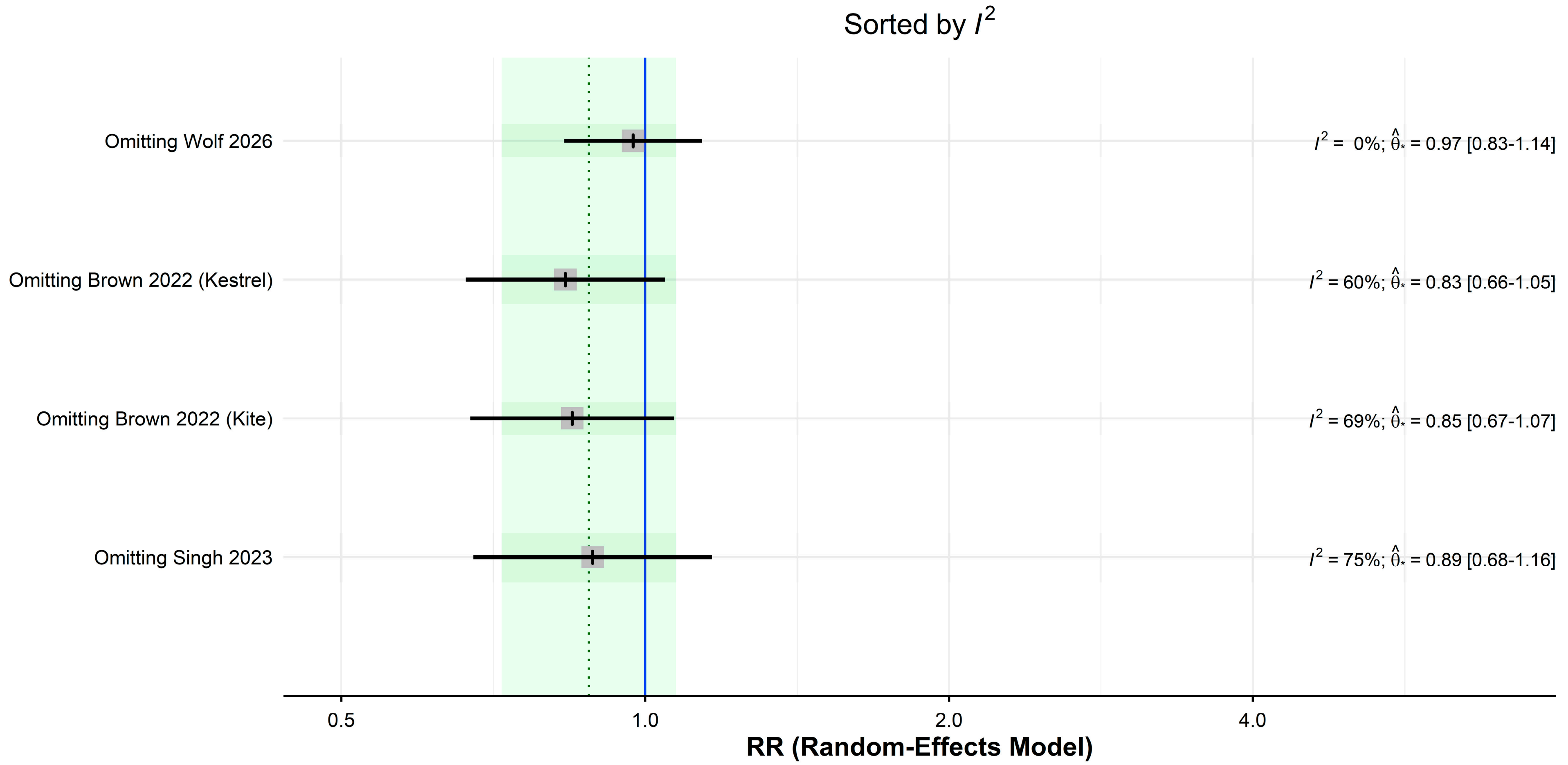

Supplementary Figure S4. Leave-one-out sensitivity analysis for at least one non-ocular adverse event.

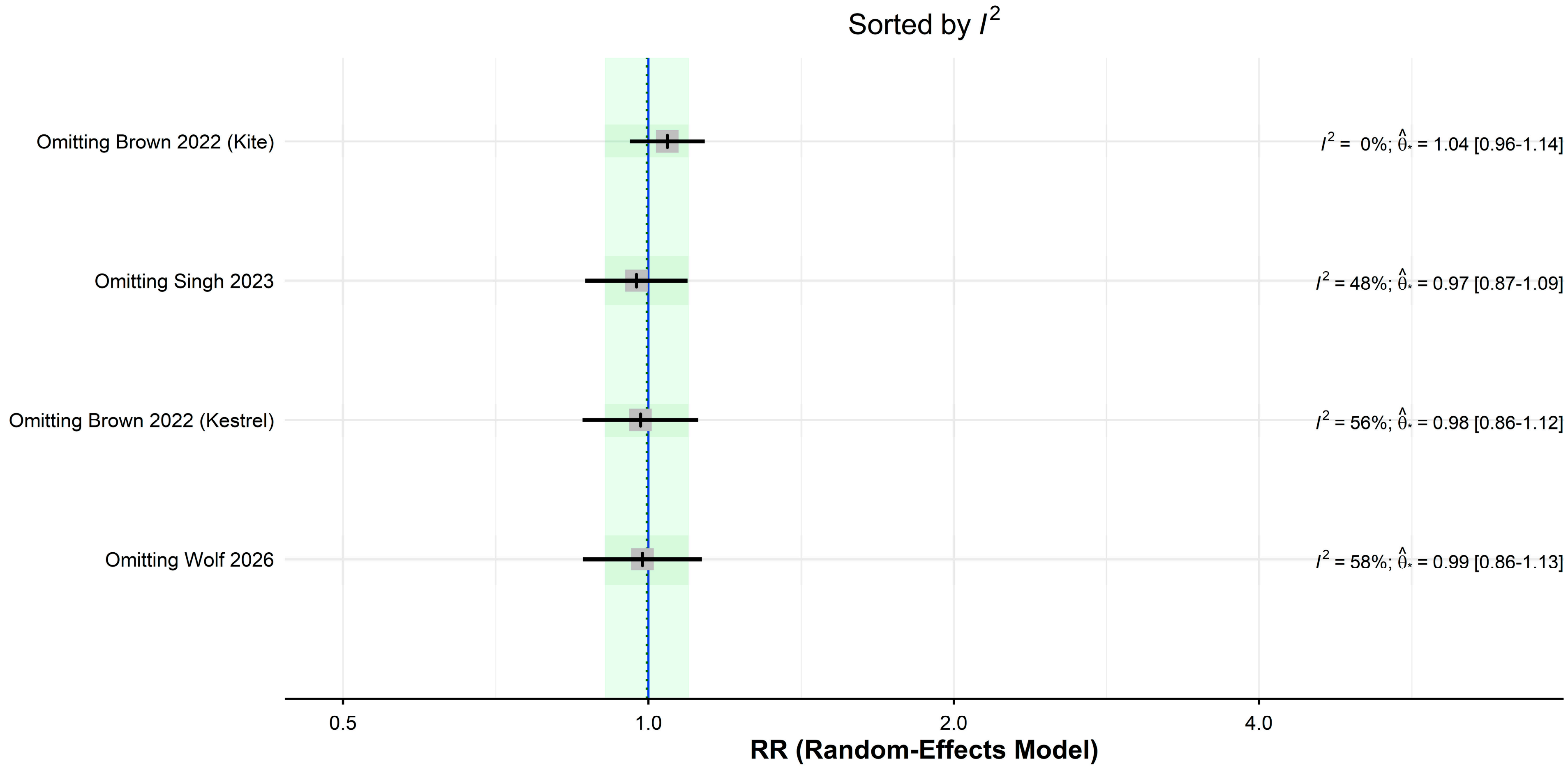

Supplementary Figure S5. Leave-one-out sensitivity analysis for serious ocular adverse events.

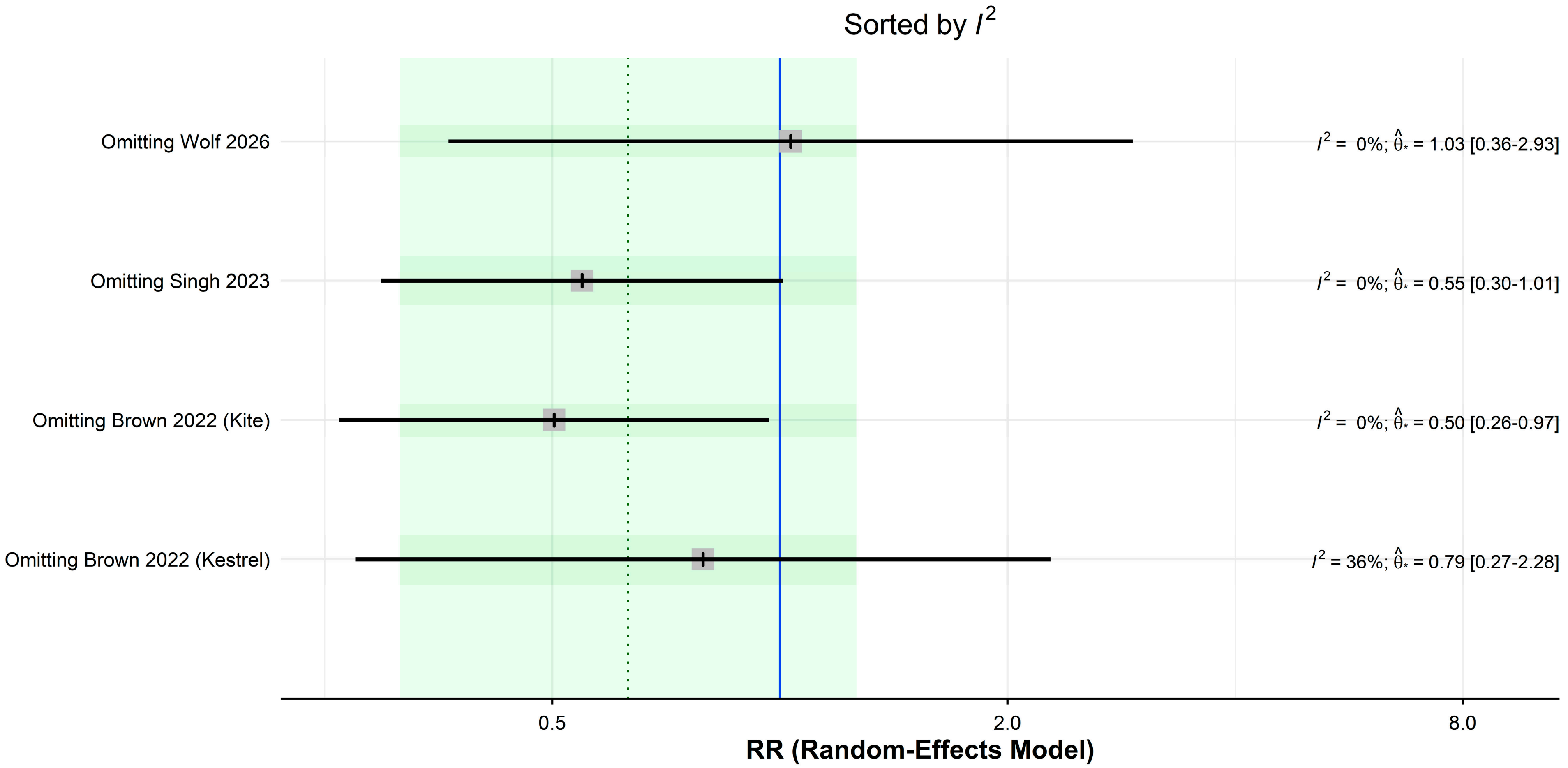

Supplementary Figure S6. Leave-one-out sensitivity analysis for serious non-ocular adverse events.

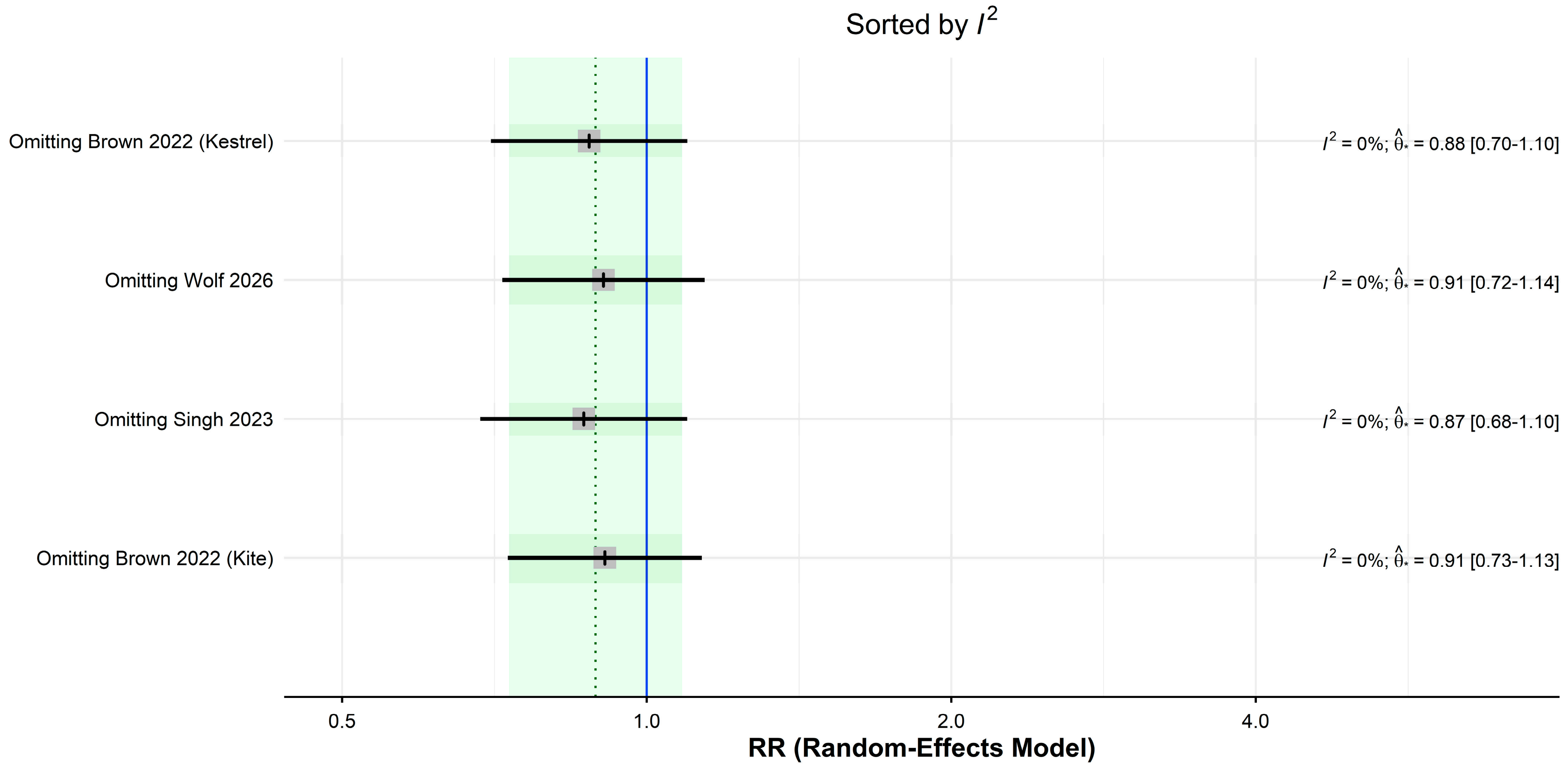

Supplementary Figure S7. Forest plot comparing intraocular inflammation between brolucizumab and control groups.

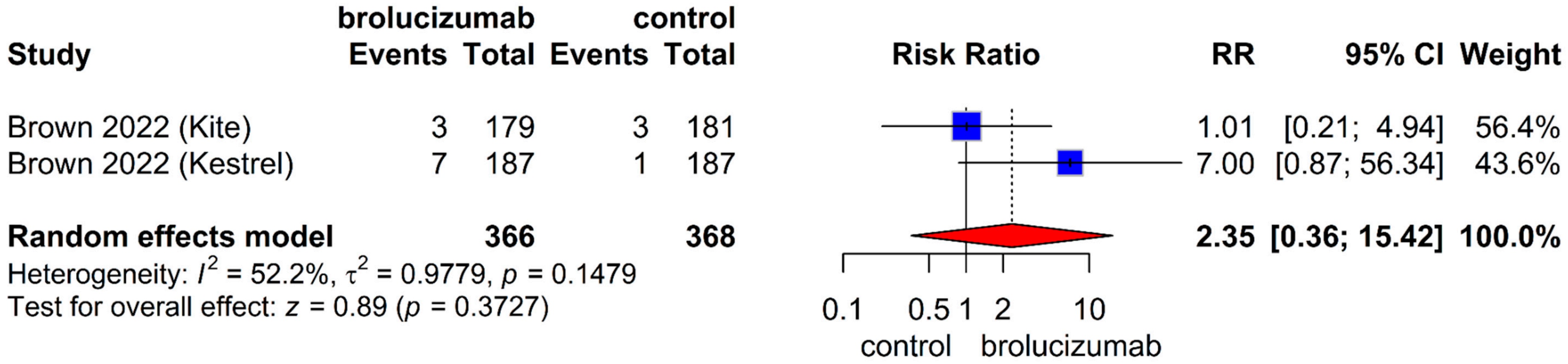

Supplementary Figure S8. Forest plot comparing retinal vasculitis between brolucizumab and control groups.

Study	brolucizumab Events	brolucizumab Total	control Events	control Total	RR	95% CI	Weight
Singh 2023	1	346	0	171	1.48	[0.06; 36.26]	50.0%
Brown 2022 (Kestrel)	1	189	0	187	2.97	[0.12; 72.40]	50.0%
**Random effects model**		**535**		**358**	**2.10**	**[0.22; 20.10]**	**100.0%**

Heterogeneity: $I^2 = 0.0\%$, $\tau^2 = 0$, $p = 0.7638$
Test for overall effect: $z = 0.64$ ($p = 0.5199$)

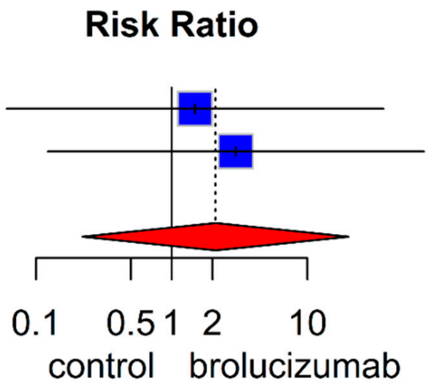

Supplementary Figure S9. Forest plot comparing retinal vascular occlusion between brolucizumab and control groups.

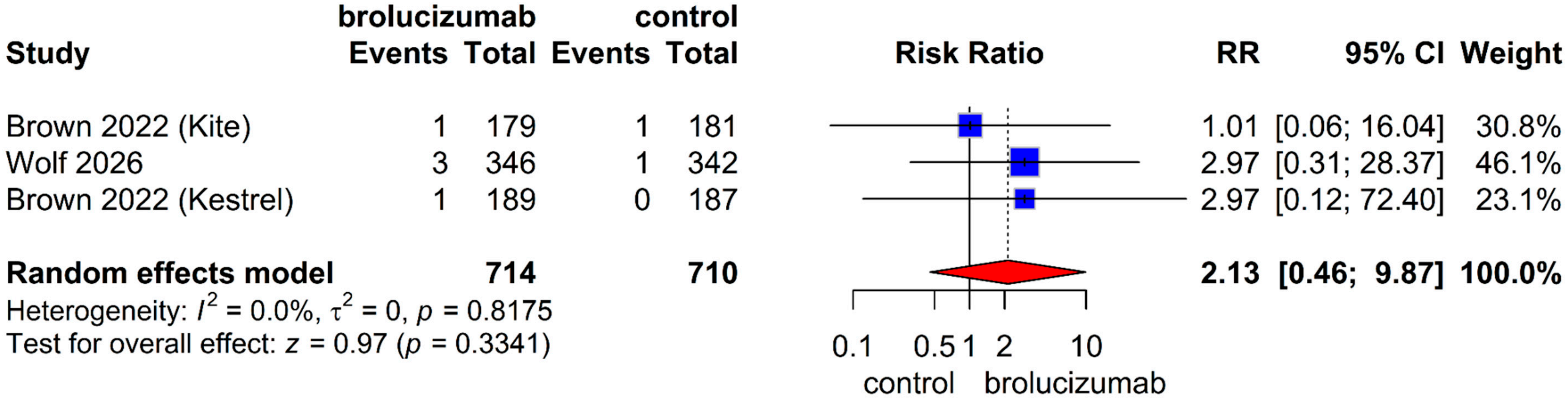

Supplementary Figure S10. Leave-one-out sensitivity analysis for retinal vascular occlusion.

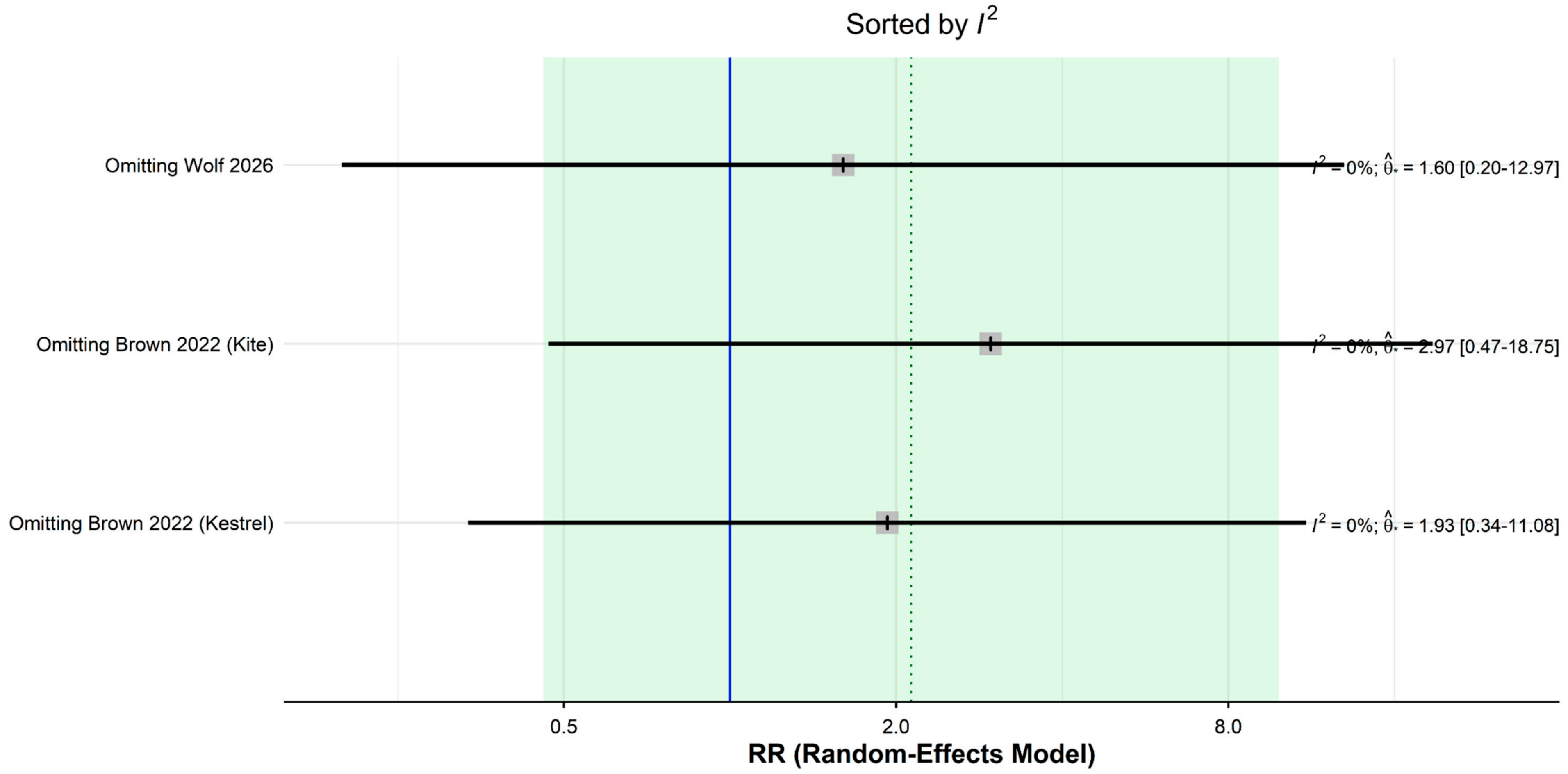

Supplementary Figure S11: Sensitivity analysis for best-corrected visual acuity by including brolucizumab 3mg dose in the analysis.

Study	brolucizumab Mean	SD	Total	control Mean	SD	Total	MD	95%-CI	Weight
Brown 2022 (Kestrel)	8.00	8.0700	379	10.50	8.7500	187	-2.50	[-3.99; -1.01]	25.6%
Brown 2022 (Kite)	10.30	8.3000	179	9.40	8.3400	181	0.90	[-0.82; 2.62]	25.0%
Singh 2023	12.24	8.9300	346	11.00	8.3700	171	1.24	[-0.33; 2.81]	25.4%
Wolf 2026	0.20	13.4000	347	-4.20	13.5000	342	4.40	[2.39; 6.41]	24.1%
**Random effects model**			**1251**			**881**	**0.96**	**[-1.79; 3.71]**	**100.0%**
**Prediction interval**								**[-8.63; 10.55]**	

Heterogeneity: $I^2 = 90.3\%$, $\tau^2 = 7.1127$, $p < 0.0001$

Test for overall effect: $z = 0.68$ ($p = 0.4935$)

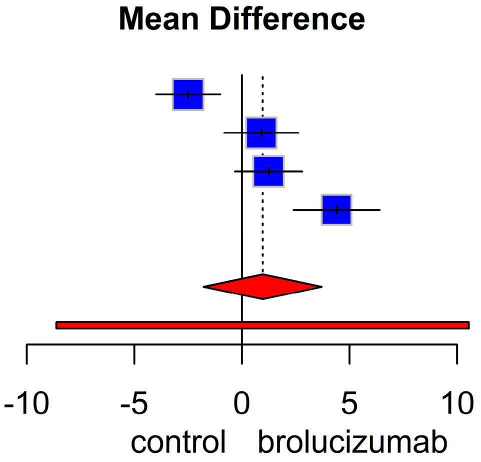

Supplementary Figure S12: Sensitivity analysis for central subfield thickness/central subfield foveal thickness by including brolucizumab 3mg dose in the analysis.

Study	MD	SE(MD)	MD	95%-CI	Weight
Singh 2023	-41.4000	8.9000	-41.40	[-58.84; -23.96]	34.9%
Wykoff 2022 (Kite)	-29.2000	11.4000	-29.20	[-51.54; -6.86]	30.9%
Wykoff 2022 (Kestrel)	-2.9000	9.3000	-2.90	[-21.13; 15.33]	34.2%
**Random effects model**			**-24.45**	**[-47.43; -1.47]**	**100.0%**
**Prediction interval**				**[-115.98; 67.08]**	

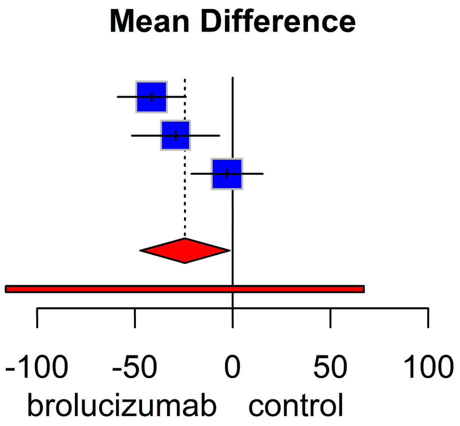

Heterogeneity: I^2 = 78.2%, τ^2 = 315.0401, p = 0.0102
Test for overall effect: z = -2.09 (p = 0.0370)

Supplementary Figure S13: Sensitivity analysis for at least one ocular adverse event by including brolucizumab 3mg dose in the analysis.

Study	brolucizumab Events	brolucizumab Total	control Events	control Total	RR	95%-CI	Weight
Wolf 2026	119	347	168	342	0.70	[0.58; 0.84]	29.5%
Singh 2023	105	346	59	171	0.88	[0.68; 1.14]	23.7%
Brown 2022 (Kite)	53	179	52	181	1.03	[0.75; 1.42]	19.8%
Brown 2022 (Kestrel)	157	379	73	187	1.06	[0.86; 1.32]	27.0%
**Random effects model**		**1251**		**881**	**0.89**	**[0.73; 1.10]**	**100.0%**
**Prediction interval**						**[0.47; 1.69]**	

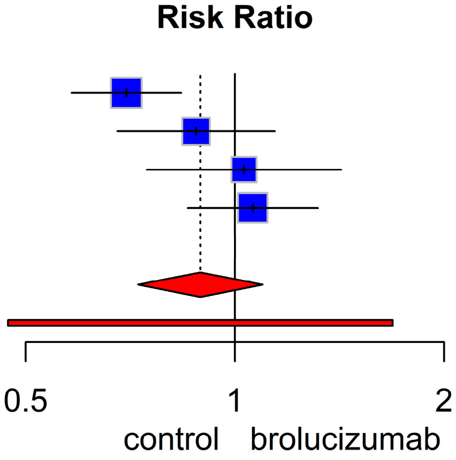

Heterogeneity: $I^2 = 69.9\%$, $\tau^2 = 0.0290$, $p = 0.0187$

Test for overall effect: $z = -1.09$ ($p = 0.2767$)

Supplementary Figure S14: Sensitivity analysis for at least one non-ocular adverse event by including brolucizumab 3mg dose in the analysis.

Study	brolucizumab Events	brolucizumab Total	control Events	control Total	RR	95%-CI	Weight
Brown 2022 (Kite)	108	179	127	181	0.86	[0.74; 1.00]	23.2%
Brown 2022 (Kestrel)	250	379	122	187	1.01	[0.89; 1.15]	29.2%
Wolf 2026	185	347	178	342	1.02	[0.89; 1.18]	25.5%
Singh 2023	209	346	96	171	1.08	[0.92; 1.26]	22.1%
**Random effects model**		**1251**		**881**	**0.99**	**[0.91; 1.08]**	**100.0%**
**Prediction interval**						**[0.80; 1.23]**	

Heterogeneity: $I^2 = 36.1\%$, $\tau^2 = 0.0027$, $p = 0.1955$

Test for overall effect: $z = -0.21$ ($p = 0.8335$)

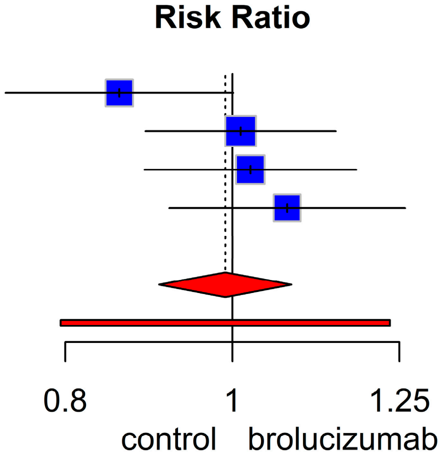

Supplementary Figure S15: Sensitivity analysis for serious ocular adverse events by including brolucizumab 3mg dose in the analysis.

Study	brolucizumab Events	brolucizumab Total	control Events	control Total	RR	95%-CI	Weight
Wolf 2026	10	347	22	342	0.45	[0.22; 0.93]	46.0%
Brown 2022 (Kestrel)	9	379	4	187	1.11	[0.35; 3.56]	27.9%
Brown 2022 (Kite)	4	179	3	181	1.35	[0.31; 5.94]	19.9%
Singh 2023	3	346	0	171	3.46	[0.18; 66.70]	6.2%
**Random effects model**		**1251**		**881**	**0.82**	**[0.38; 1.75]**	**100.0%**
**Prediction interval**						**[0.13; 5.25]**	

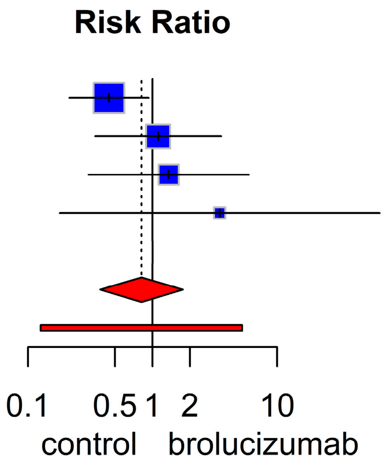

Heterogeneity: $I^2 = 23.1\%$, $\tau^2 = 0.1904$, $p = 0.2722$
Test for overall effect: $z = -0.52$ ($p = 0.6002$)

Supplementary Figure S16: Sensitivity analysis for serious non-ocular adverse events by including brolucizumab 3mg dose in the analysis.

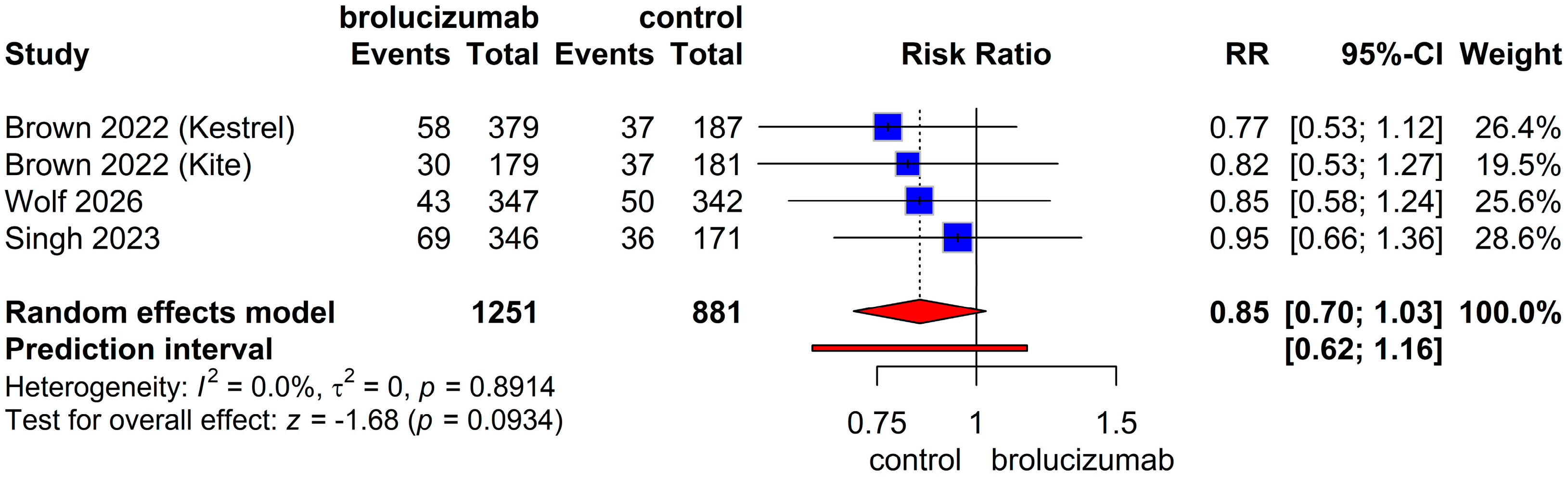

Supplementary Figure S17. Doi plot for publication bias assessment of best-corrected visual acuity.

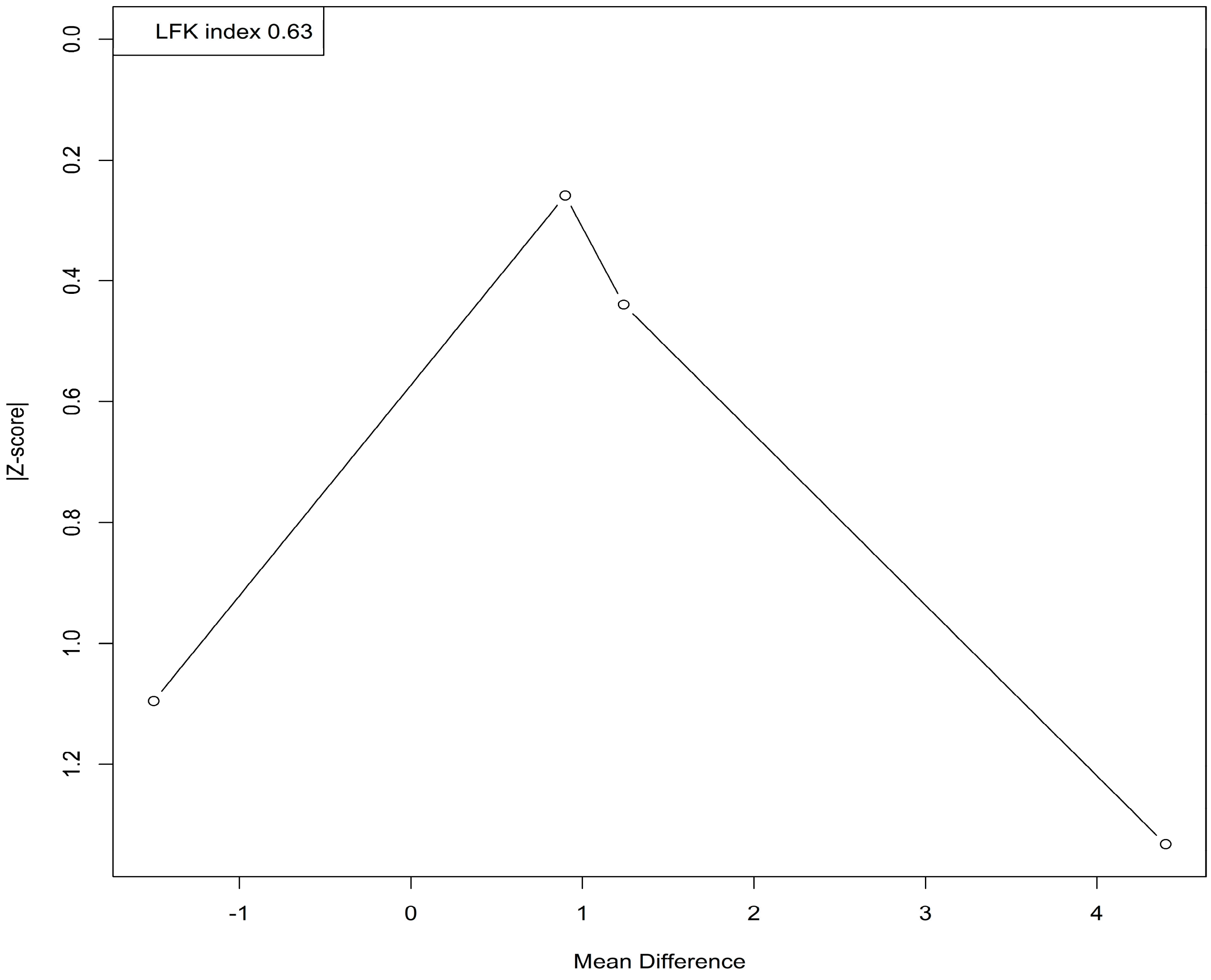

Supplementary Figure S18. Doi plot for publication bias assessment of central subfield thickness/central subfield foveal thickness.

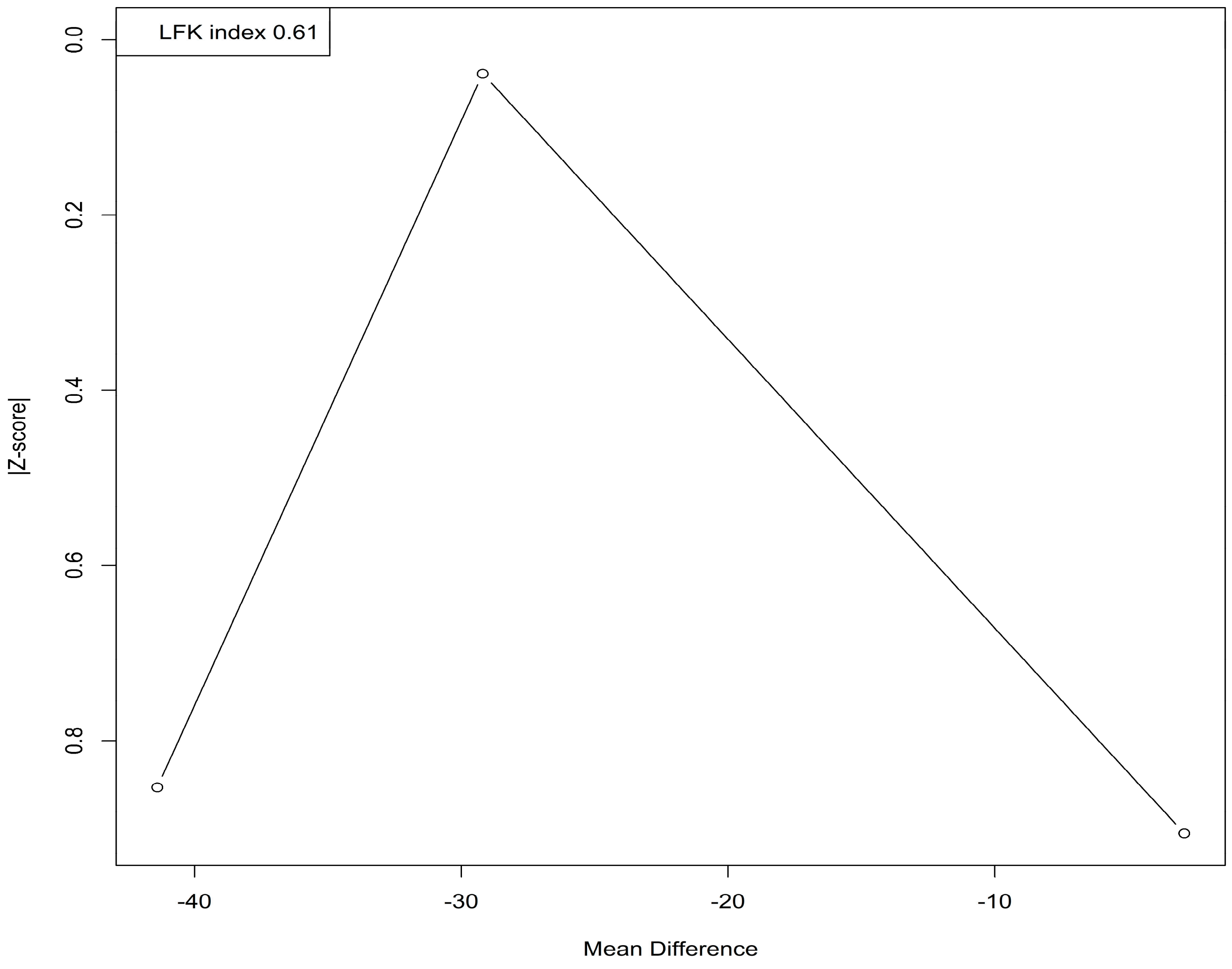

Supplementary Figure S19. Doi plot for publication bias assessment of at least one ocular adverse event.

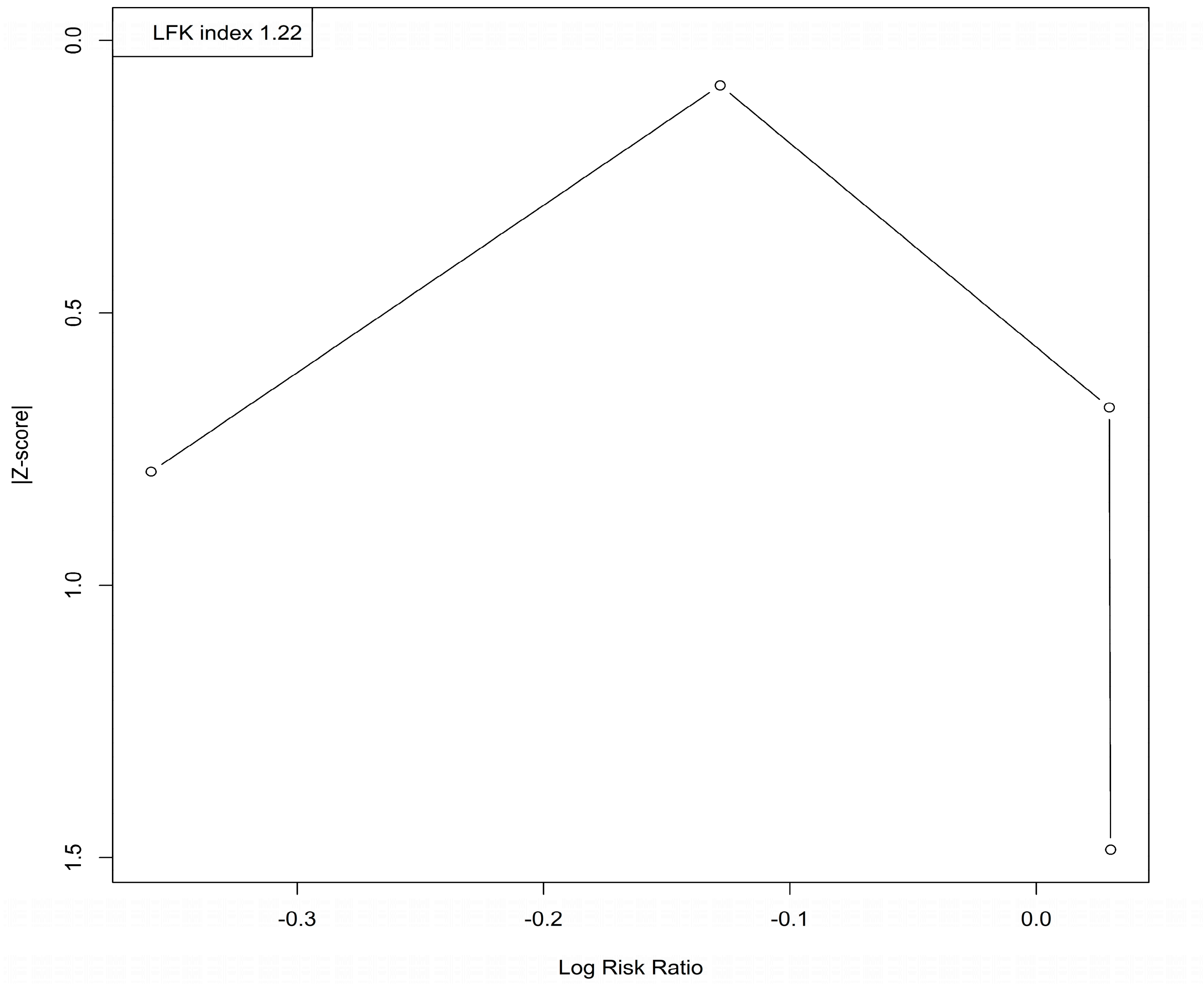

Supplementary Figure S20. Doi plot for publication bias assessment of at least one non-ocular adverse event.

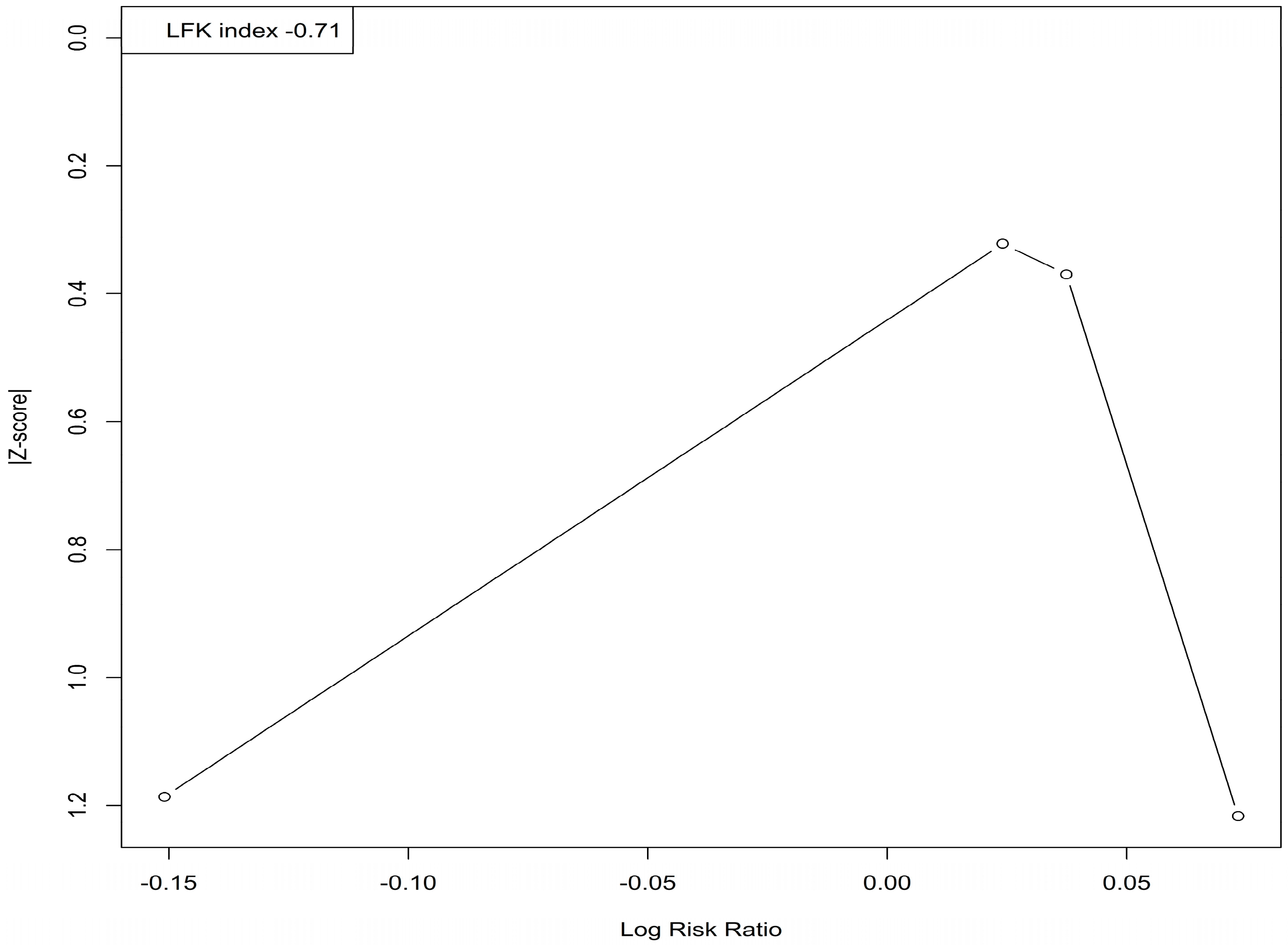

Supplementary Figure S21. Doi plot for publication bias assessment of serious non-ocular adverse events.

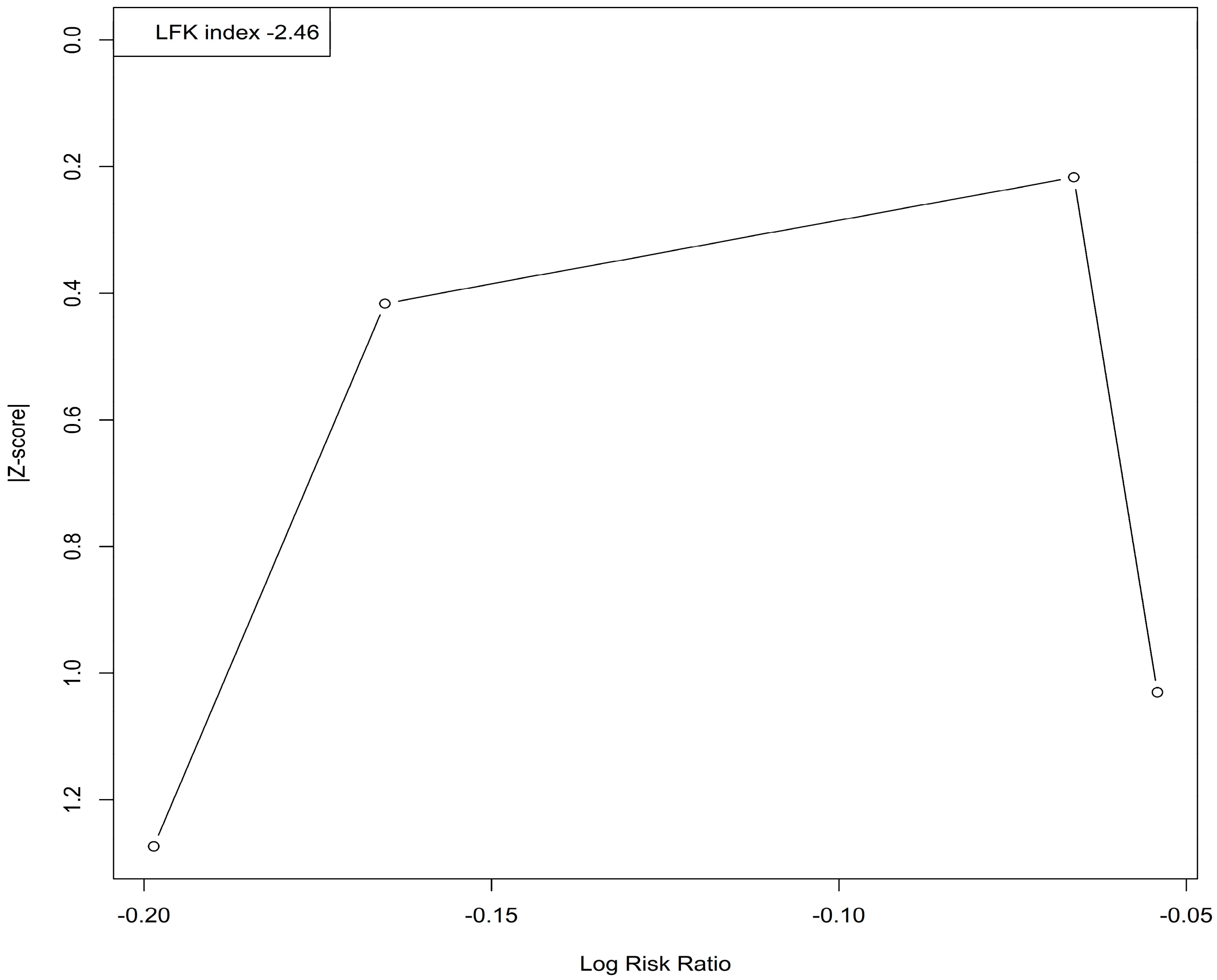

Supplementary Figure S22. Doi plot for publication bias assessment of serious ocular adverse events.

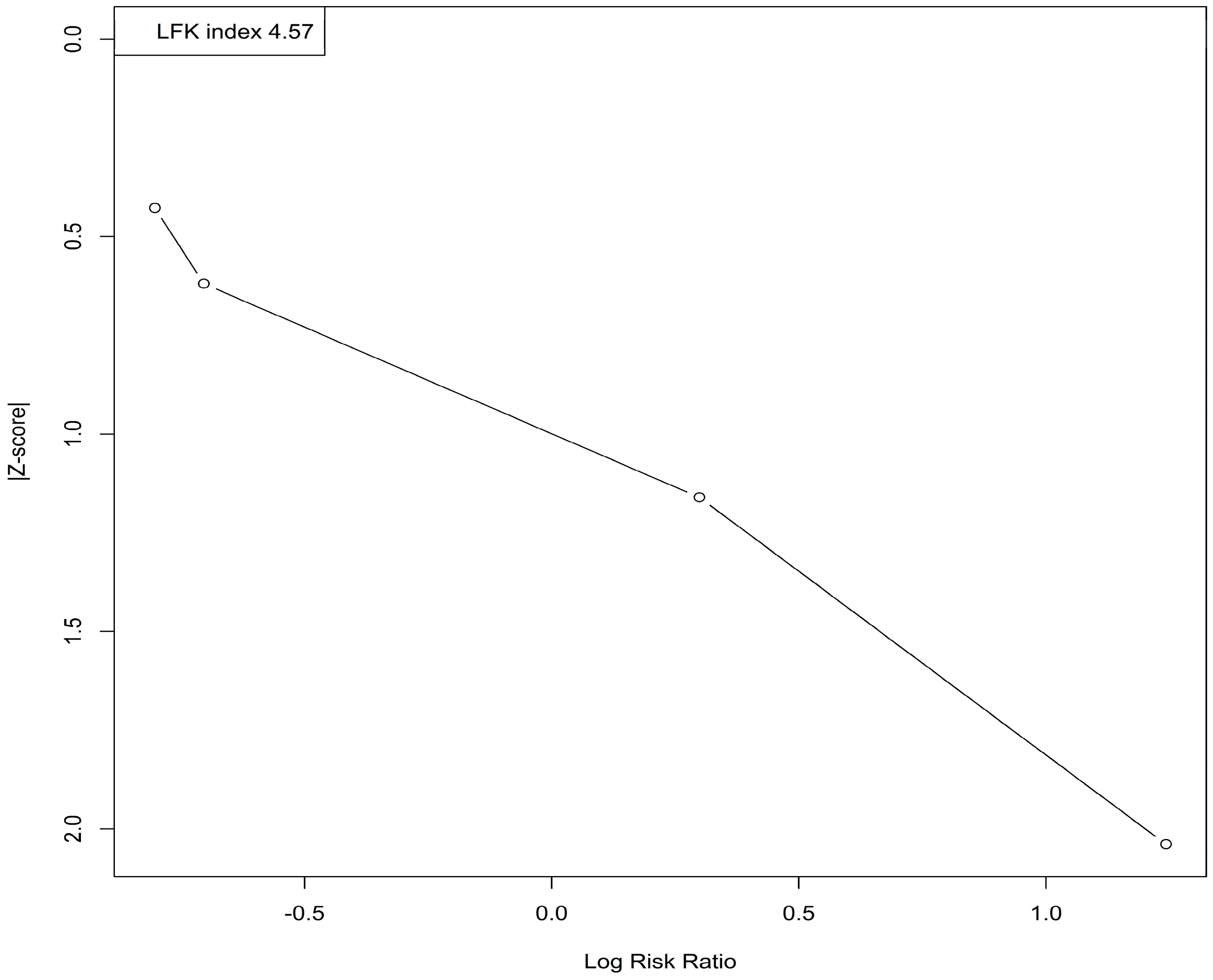

PRISMA Checklist

Section and Topic	Item #	Checklist item	Location where item is reported
**TITLE**			
Title	1	Identify the report as a systematic review.	1
**ABSTRACT**			
Abstract	2	See the PRISMA 2020 for Abstracts checklist.	3
**INTRODUCTION**			
Rationale	3	Describe the rationale for the review in the context of existing knowledge.	5-6
Objectives	4	Provide an explicit statement of the objective(s) or question(s) the review addresses.	6
**METHODS**			
Eligibility criteria	5	Specify the inclusion and exclusion criteria for the review and how studies were grouped for the syntheses.	7
Information sources	6	Specify all databases, registers, websites, organizations, reference lists and other sources searched or consulted to identify studies. Specify the date when each source was last searched or consulted.	7
Search strategy	7	Present the full search strategies for all databases, registers and websites, including any filters and limits used.	7
Selection process	8	Specify the methods used to decide whether a study met the inclusion criteria of the review, including how many reviewers screened each record and each report retrieved, whether they worked independently, and if applicable, details of automation tools used in the process.	7-8
Data collection process	9	Specify the methods used to collect data from reports, including how many reviewers collected data from each report, whether they worked independently, any processes for obtaining or confirming data from study investigators, and if applicable, details of automation tools used in the process.	8
Data items	10a	List and define all outcomes for which data were sought. Specify whether all results that were compatible with each outcome domain in each study were sought (e.g., for all measures, time points, analyses), and if not, the methods used to decide which results to collect.	8
	10b	List and define all other variables for which data were sought (e.g., participant and intervention characteristics, funding sources). Describe any assumptions made about any missing or unclear information.	8
Study risk of bias assessment	11	Specify the methods used to assess risk of bias in the included studies, including details of the tool(s) used, how many reviewers assessed each study and whether they worked independently, and if applicable, details of automation tools used in the process.	8
Effect measures	12	Specify for each outcome the effect measure(s) (e.g., risk ratio, mean difference) used in the synthesis or presentation of results.	9
Synthesis methods	13a	Describe the processes used to decide which studies were eligible for each synthesis (e.g., tabulating the study intervention characteristics and comparing against the planned groups for each synthesis (item #5)).	7-8
	13b	Describe any methods required to prepare the data for presentation or synthesis, such as handling of missing summary statistics, or data conversions.	8
	13c	Describe any methods used to tabulate or visually display results of individual studies and syntheses.	
	13d	Describe any methods used to synthesize results and provide a rationale for the choice(s). If meta-analysis was performed, describe the model(s), method(s) to identify the presence and extent of statistical heterogeneity, and software package(s) used.	9
	13e	Describe any methods used to explore possible causes of heterogeneity among study results (e.g., subgroup analysis, meta-regression).	

Section and Topic	Item #	Checklist item	Location where item is reported
	13f	Describe any sensitivity analyses conducted to assess robustness of the synthesized results.	9
Reporting bias assessment	14	Describe any methods used to assess risk of bias due to missing results in a synthesis (arising from reporting biases).	9
Certainty assessment	15	Describe any methods used to assess certainty (or confidence) in the body of evidence for an outcome.	8
**RESULTS**			
Study selection	16a	Describe the results of the search and selection process, from the number of records identified in the search to the number of studies included in the review, ideally using a flow diagram.	10
	16b	Cite studies that might appear to meet the inclusion criteria, but which were excluded, and explain why they were excluded.	10
Study characteristics	17	Cite each included study and present its characteristics.	10
Risk of bias in studies	18	Present assessments of risk of bias for each included study.	10-11
Results of individual studies	19	For all outcomes, present, for each study: (a) summary statistics for each group (where appropriate) and (b) an effect estimate and its precision (e.g., confidence/credible interval), ideally using structured tables or plots.	
Results of syntheses	20a	For each synthesis, briefly summarize the characteristics and risk of bias among contributing studies.	10-13
	20b	Present results of all statistical syntheses conducted. If meta-analysis was done, present for each the summary estimate and its precision (e.g., confidence/credible interval) and measures of statistical heterogeneity. If comparing groups, describe the direction of the effect.	11-12
	20c	Present results of all investigations of possible causes of heterogeneity among study results.	
	20d	Present results of all sensitivity analyses conducted to assess the robustness of the synthesized results.	11-12
Reporting biases	21	Present assessments of risk of bias due to missing results (arising from reporting biases) for each synthesis assessed.	12
Certainty of evidence	22	Present assessments of certainty (or confidence) in the body of evidence for each outcome assessed.	13
**DISCUSSION**			
Discussion	23a	Provide a general interpretation of the results in the context of other evidence.	14-16
	23b	Discuss any limitations of the evidence included in the review.	17-18
	23c	Discuss any limitations of the review processes used.	17-18
	23d	Discuss implications of the results for practice, policy, and future research.	16-18
**OTHER INFORMATION**			
Registration and protocol	24a	Provide registration information for the review, including register name and registration number, or state that the review was not registered.	7
	24b	Indicate where the review protocol can be accessed, or state that a protocol was not prepared.	7

Section and Topic	Item #	Checklist item	Location where item is reported
	24c	Describe and explain any amendments to information provided at registration or in the protocol.	7
Support	25	Describe sources of financial or non-financial support for the review, and the role of the funders or sponsors in the review.	20
Competing interests	26	Declare any competing interests of review authors.	20
Availability of data, code and other materials	27	Report which of the following are publicly available and where they can be found: template data collection forms; data extracted from included studies; data used for all analyses; analytic code; any other materials used in the review.	20
